# The integrin protein ITGβ1 effectively suppresses porcine epidemic diarrhea virus replication through facilitating MDA5 oligomerization and subsequent activation of the type I interferon signaling pathway

**DOI:** 10.1128/jvi.01553-25

**Published:** 2025-10-17

**Authors:** Jiarong Yu, Qi Sun, Junrui Zhu, Yuxi Cui, Pengfei Chen, Wei Shen, Ying Yue, YiFeng Jiang, Changlong Liu, Huili Liu, Guangzhi Tong, Fei Gao, Yanjun Zhou

**Affiliations:** 1 Shanghai Veterinary Research Institute, Chinese Academy of Agricultural Sciences 118161, Shanghai, China; 2 China Animal Health and Epidemiology Center 499141 , Qingdao, Shandong, China; 3 Shanghai Academy of Agricultural Sciences, Institute of Animal Husbandry and Veterinary Medicine 74594, Shanghai, China; 4 Jiangsu Co-Innovation Center for the Prevention and Control of Important Animal Infectious Diseases and Zoonoses, Yangzhou University 38043, Yangzhou, China; University of Kentucky College of Medicine, Lexington, Kentucky, USA

**Keywords:** ITGβ1, PEDV, IFN-I, MDA5

## Abstract

Integrins are cell surface adhesion molecules. They bridge the intracellular and extracellular environments, enabling bidirectional transmembrane signaling and regulating immune responses. However, it remains unclear whether integrin protein β1 (ITGβ1) is involved in innate immune responses. In our previous study, we demonstrated that porcine epidemic diarrhea virus (PEDV) can induce type I interferon (IFN-I) production. In this study, we observed that ITGβ1 expression is rapidly induced following PEDV infection and further established that PEDV infection primarily promoted ITGβ1 expression through upregulation of the transcription factor c-Myc. We hypothesized that ITGβ1 might be involved in PEDV-induced innate immune responses through IFN-I production. Our investigation revealed ITGβ1 overexpression promotes the phosphorylation and subsequent nuclear translocation of both interferon regulatory factor 3 (IRF3) and NF-κB, thereby enhancing SeV-induced IFN-β promoter activity. Furthermore, we showed that ITGβ1 functions as an activator in the melanoma differentiation-associated protein 5 (MDA5)-mediated IFN-I signaling pathway. More importantly, we demonstrated that ITGβ1 is critically involved in PEDV-induced IFN-I antiviral responses. Mechanistically, ITGβ1 facilitates MDA5 oligomerization by specifically interacting with its caspase activation and recruitment domain (CARD), thereby enhancing dsRNA-recruitment capacity. In summary, the findings of this study indicate that ITGβ1 acts as an activator of the MDA5-dependent IFN-I antiviral innate immune response and positively regulates the MDA5-mediated RIG-I-like receptor signaling pathway.

**IMPORTANCE:**

Porcine epidemic diarrhea virus (PEDV), an alpha coronavirus, severely impacts newborn piglets, leading to acute manifestations including vomiting, diarrhea, dehydration, and high mortality rates in suckling piglets. These consequences have devastating implications for the global swine industry. Within the host’s innate antiviral response, RIG-I-like receptors (RLRs) are critical for the activation of the interferon signaling pathway. Integrin proteins, known for their role in regulating bidirectional signal transduction across the cell membrane, are associated with numerous viral infections. In this study, utilizing PEDV as an infection model, we demonstrated that overexpression of ITGβ1 suppresses PEDV replication, while knockdown of ITGβ1 expression enhances it. Additionally, ITGβ1 significantly augments PEDV-induced type I interferon production in host cells. We further elucidated that ITGβ1 interacts with the 2CARD region of MDA5, promoting MDA5 oligomerization and the transmission of activation signals. These findings establish ITGβ1 as a positive regulatory factor in MDA5-mediated RLR signaling pathway. These findings not only identify ITGβ1 as a novel host antiviral protein against PEDV but also reveal, for the first time, a previously unrecognized function of ITGβ1 in the cellular innate antiviral immune response.

## INTRODUCTION

Integrins are a type of class I transmembrane glycoprotein, consisting of 18 α subunits and 8 β subunits (1). It is composed of three distinct domains: the extracellular domain, the transmembrane domain, and the cytoplasmic tail. Notably, the extracellular domain is the largest, ranging from approximately 80 to 150 kDa, while the cytoplasmic tail is relatively shorter, consisting of 10–70 amino acids. Through various forms of assembly, α and β subunits can form 24 distinct heterodimers, which play crucial roles in regulating multiple cellular functions. These include adhesion to the extracellular matrix, modulation of cell trafficking, and facilitation of bidirectional signal transduction (2–4). Intriguingly, to date, integrin proteins have been identified as receptors or co-receptors for a variety of viruses, or are exploited by viruses for their adhesive and signal transduction properties to facilitate viral entry into host cells. Examples include the foot-and-mouth disease virus (5), reovirus (6), rotavirus (7), vaccinia virus (VV) (8), human cytomegalovirus (HCMV) (9), Ebola virus (EboV) (10), and SARS-CoV-2 (11). These viruses utilize either the RGD (Arg-Gly-Asp) tripeptide motif or non-RGD binding mechanisms to interact with various integrin proteins, thereby promoting adhesion, cytoskeleton rearrangement, integrin activation, and enhanced intracellular signaling, which collectively facilitate viral infection of host cells. Additionally, as key components bridging the intracellular and extracellular environments, integrins play a significant role in modulating immune responses. Recent studies have revealed that integrin αxβ2 (CD11c/CD18 and CR4) acts as an effective inducer of early antiviral immunity, controlling viremia during the acute phase of HIV-1 infection through CR4, while integrin αMβ2 (CD11b/CD18 and CR3) downregulates. Type I interferon (IFN-I) during the chronic phase of HIV-1 infection (12, 13). Integrin αM (CD11b) in monocytes has been recognized as a non-Toll-like receptor (TLR), pattern recognition receptor (PRR), and a regulator of innate immune responses (14). The gH/gL glycoproteins of herpes simplex virus (HSV) bind to integrin αvβ3, inducing the production of IFN-I and activation of NF-κB (15). These studies highlight the antiviral potential of integrins. However, there is still a lack of direct evidence demonstrating how integrin proteins participate in antiviral processes, and the mechanisms by which they regulate innate immune response remain poorly understood.

Porcine epidemic diarrhea virus (PEDV) is an enveloped, single-stranded, positive-sense RNA virus with a genome approximately 28 kb in length. It belongs to the order Nidovirales, family Coronaviridae, and genus *Alphacoronavirus* (16). PEDV infection primarily causes acute watery diarrhea, vomiting, dehydration, and emaciation in newborn piglets, with morbidity and mortality rates reaching up to 100% in piglets within 10 days of age (17–19). In late 2010, highly pathogenic variants of PEDV characterized by genetic mutations emerged and spread globally, causing significant economic losses to the swine industry worldwide (20, 21). During the early stages of infection, the virus triggers the host’s antiviral innate immune response to counteract viral invasion, primarily through the secreting of IFN-Is and inflammatory cytokines to limit viral replication. In contrast, PEDV can suppress the host’s innate immune response by encoding specific viral proteins, such as nsp1, nsp3, nsp7, nsp14, nsp15, nsp16, and the nucleocapsid (N) protein (22). Our previous work revealed that the expression of integrin β1 (ITGβ1) is significantly upregulated following PEDV infection. However, whether ITGβ1, as a cell surface molecule, participates in the innate immune response against the virus, and the relationship between the high expression of ITGβ1 in host cells and the PRR-triggered innate immune response, remains unclear. Further investigation into the functional role of ITGβ1 may provide novel insights into anti-PEDV infection strategies.

In this study, we discovered that PEDV infection upregulates ITGβ1 expression primarily through the manipulation of the transcription factor c-Myc. Notably, the increased expression of ITGβ1 was shown to suppress PEDV replication, confirming its role as an activator in the melanoma differentiation-associated protein 5 (MDA5)-mediated IFN-I signaling pathway. Furthermore, our findings indicated that ITGβ1 positively modulates the innate antiviral immune response. These results establish a novel link between ITGβ1 and the PRR MDA5, provide new insights into the ITGβ1-mediated signaling cascade, and suggest a promising antiviral strategy for protecting against PEDV infection.

## RESULTS

### PEDV infection upregulates ITGβ1 expression

**Fig 1:**
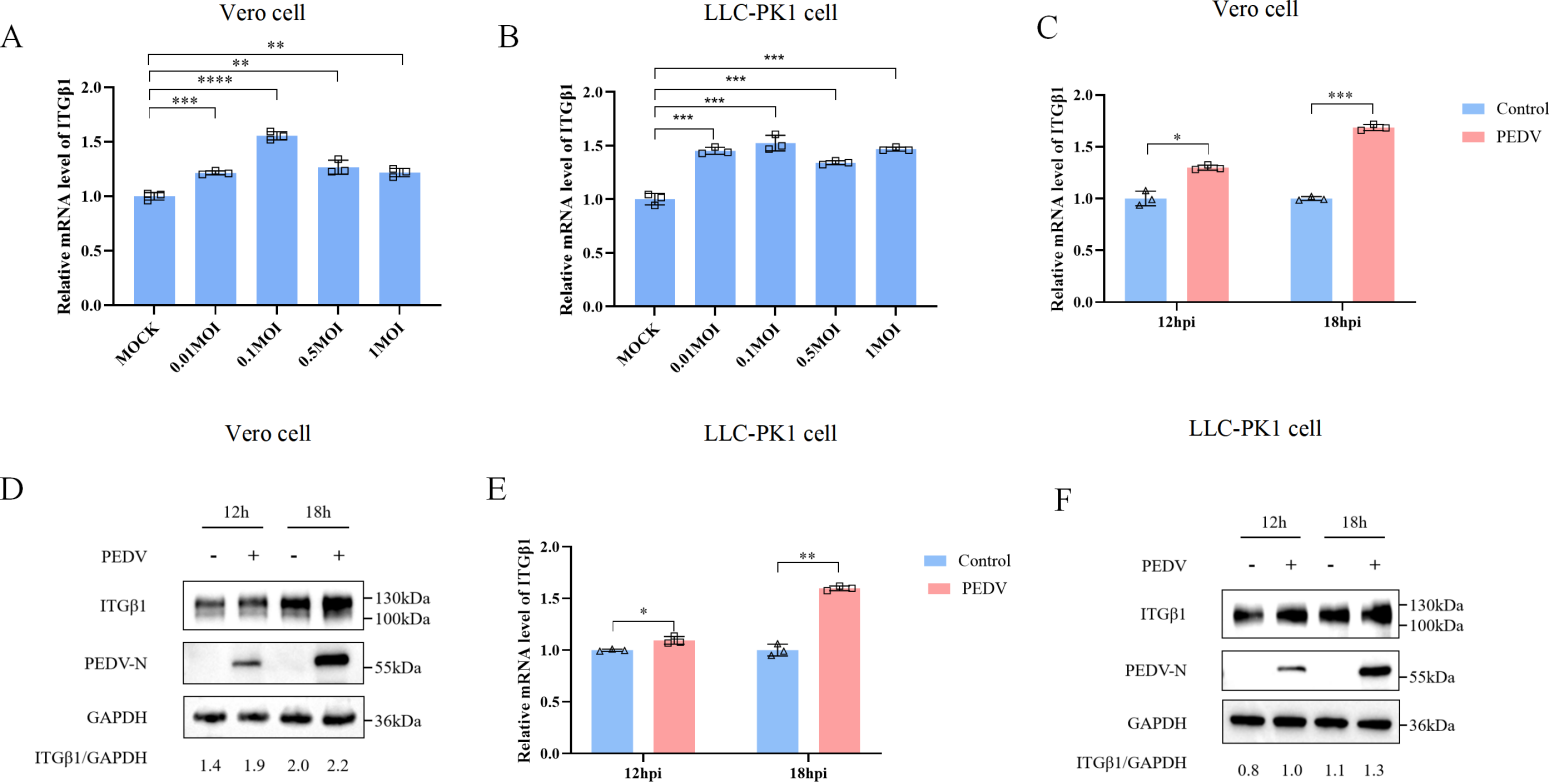
PEDV infection upregulates ITGβ1 expression in both Vero and LLC-PK1 cells. (**A and B**) qPCR was used to measure the expression levels of ITGβ1 mRNA in Vero and LLC-PK1 cells infected with PEDV at different MOIs (0.01, 0.1, 0.5, and 1). (**C and D**) Vero cells were infected with PEDV at an MOI of 0.1 or mock-infection, and ITGβ1 expression was analyzed at 12 and 18 hpi. (**E and F**) Similarly, qPCR and Western blotting analysis were performed to assess the ITGβ1 expression in LLC-PK1 cells. Data are presented as means ± SD from three independent experiments. *, *P*  <  0.05; **, *P*  <  0.01; ***, *P*  <  0.001; ****, *P* < 0.0001 by two-tailed Student’s *t*-test (for two-group comparisons) or one-way ANOVA (for multi-group comparisons).

Integrins typically exploit their ability to bridge intracellular and extracellular environments to facilitate viral infections. To investigate whether PEDV infection affects ITGβ1 expression, Vero and LLC-PK1 cells were infected with the PEDV G2 subgroup strain FJzz1 at multiplicities of infection (MOIs) of 0.01, 0.1, 0.5, and 1. Cell samples were harvested 24 h post-infection (hpi), and total RNA was extracted for quantitative real-time PCR (qPCR) analysis of ITGβ1 expression. Our results demonstrated ITGβ1 mRNA levels were significantly increased in both cell types across all infection doses compared to the uninfected controls (Fig. 1A and B). Subsequently, cells infected with 0.1 MOI PEDV were analyzed at 12 and 18 hpi using RT-qPCR and Western blotting. Both mRNA and protein levels of ITGβ1 were significantly elevated in PEDV-infected Vero and LLC-PK1 cells compared to controls, confirming that PEDV infection upregulates intracellular ITGβ1 expression (Fig. 1C through F).

### PEDV infection upregulates ITGβ1 expression via c-Myc

**Fig 2:**
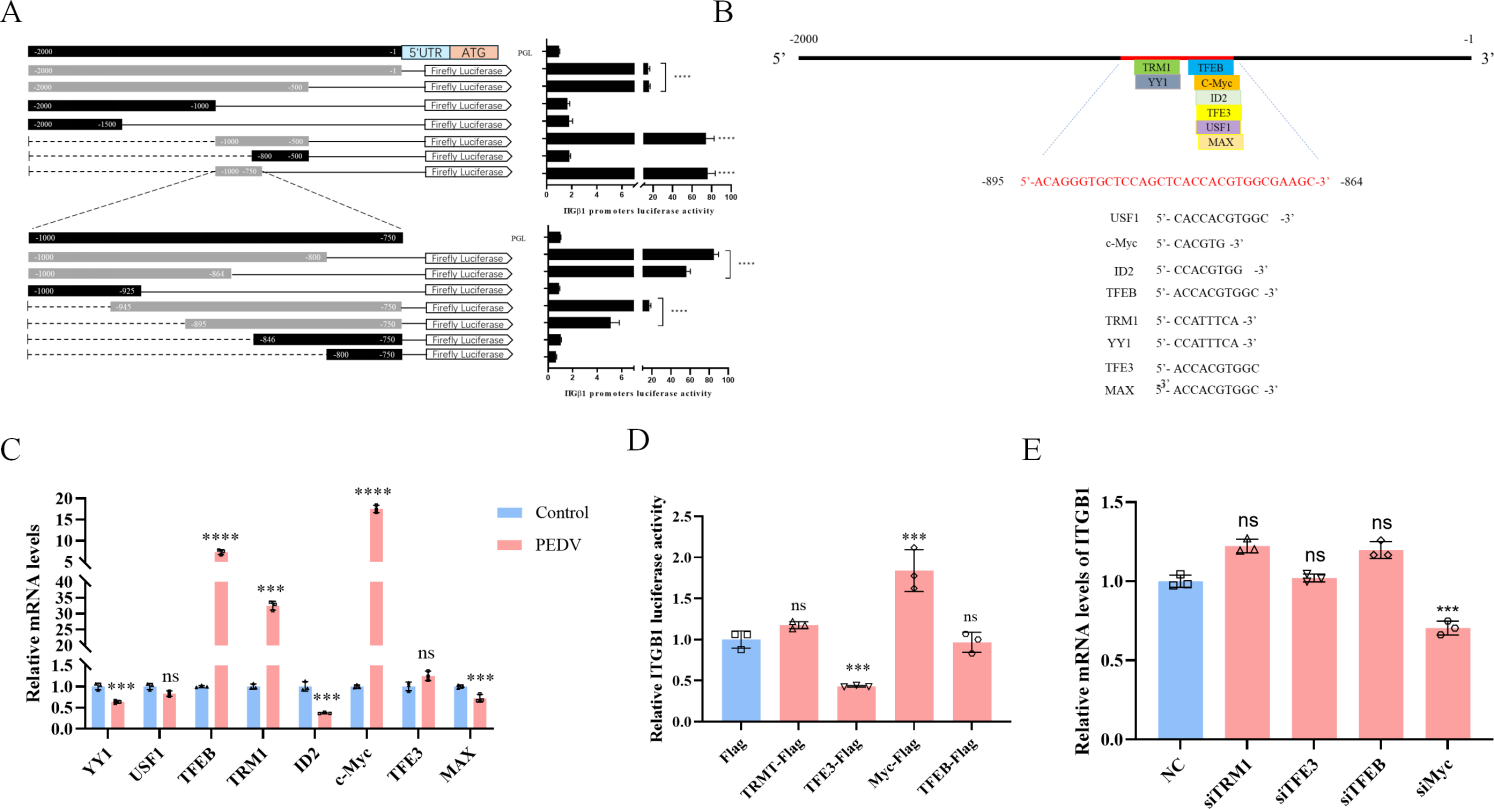
PEDV infection upregulates the expression of ITGβ1 through the mediation of the transcription factor c-Myc. (**A**) HEK293T cells were co-transfected with a series of truncated ITGβ1 promoter plasmids (−2,000 to −1) and the pRL-TK-Luc reporter plasmid, and dual-luciferase activity was analyzed 24 h post-transfection. In the figure, gray represents gene fragments with detectable luciferase activity in the luciferase activity assay, while black indicates gene fragments for which no luciferase activity was detected. (**B**) Regulatory factors within the ITGβ1 promoter region were predicted using the JASPAR vertebrate database (https://jaspar.genereg.net/). (**C**) LLC-PK1 cells were infected with PEDV at an MOI of 0.1 or mock-infected, and the mRNA expression levels of the predicted regulatory factors were detected using qPCR. (**D**) HEK293T cells were co-transfected with the ITGβ1 promoter-driven luciferase vector, plasmids expressing TRMT, TFEB, c-Myc, and TFEB, and the pRL-TK-Luc Renilla luciferase reporter vector. Dual-luciferase activity was measured 24 h post-transfection. (**E**) The expression of ITGβ1 mRNA in LLC-PK1 cells transfected with siRNAs targeting the predicted regulatory factors was assessed using qPCR. Data are shown as means ± SD of triplicate samples. ***, *P*  <  0.001; ****, *P* < 0.0001; ns, not significant by two-tailed Student’s *t*-test.

Transcription factors typically regulate gene transcription and promote protein expression by binding to specific sequences within gene promoters. Therefore, we hypothesize that during PEDV infection, host cells might modulate ITGβ1 expression through the involvement of certain transcription factors. To identify the transcription factors responsible for regulating ITGβ1 gene expression, we initially cloned the −1 to −2,000 nt region of the ITGβ1 gene, which contains the promoter sequence, into a luciferase reporter vector (pGL3-Basic). Additionally, we generated truncated gene clones of varying lengths targeting this promoter region (Fig. 2A). These constructs were then transfected into HEK293T cells, and the induced luciferase activity was measured. The results showed that the gene region containing nucleotides −895 to −864 nt induced significantly higher luciferase activity compared to the full −1 to −2000 nt region. In contrast, constructs lacking the −895 to −864 nt region exhibited negligible or low luciferase activity. These findings indicate that the core promoter region of ITGβ1 is located within the −895 to −864 nt range (Fig. 2B). To pinpoint transcription factor binding sites within the ITGβ1 promoter region, we employed the JASPAR database (http://jaspar.genereg.net/) to predict potential transcription factors that could bind to the core ITGβ1 promoter region (23). Our analysis revealed eight high-scoring candidate binding sites for transcription factors, including USF1, c-Myc, ID2, TFEB, TRM1, YY1, TFE3, and MAX, within the −895 to −864 nt core region (Fig. 2C). To identify transcription factors involved in regulating ITGβ1 expression, we infected LLC-PK1 cells with PEDV at an MOI of 0.1, harvested cell samples 24 hpi, and quantified changes in transcription factor mRNA levels using qPCR. The results demonstrated that, compared to the mock-infected group, the mRNA levels of TFEB, TRM1, c-Myc, and TFE3 were upregulated in PEDV-infected cells, consistent with the previously observed upregulation trend of ITGβ1 mRNA (Fig. 2C). We further constructed eukaryotic expression vectors (pCMV-3X Flag) for TFEB, TRM1, c-Myc, and TFE3 and co-transfected them with the luciferase reporter plasmid containing the ITGβ1 promoter region into HEK293T cells. Luciferase activity assays revealed that only the transcription factor c-Myc significantly enhanced ITGβ1 luciferase activity (Fig. 2D). Similarly, using specific siRNAs targeting TFEB, TRM1, c-Myc, and TFE3 for validation, qPCR analysis demonstrated a significant reduction in ITGβ1 mRNA levels exclusively in LLC-PK1 cells transfected with sic-Myc (Fig. 2E). These findings collectively indicate that during PEDV infection, host cells predominantly upregulate ITGβ1 expression through the transcription factor c-Myc.

### Overexpression of ITGβ1 effectively inhibits PEDV replication in both Vero and LLC-PK1 cells

**Fig 3:**
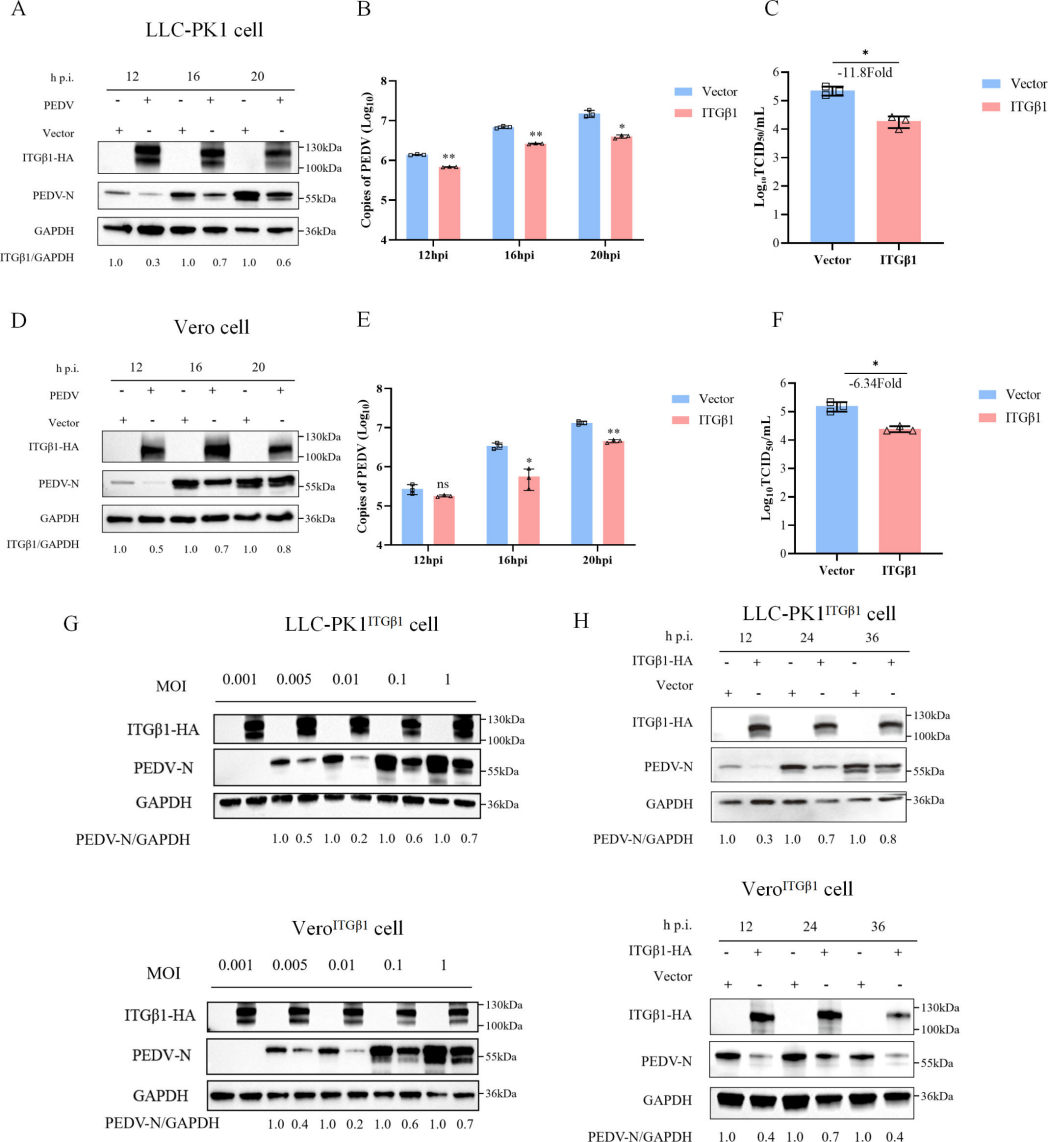
Overexpression of ITGβ1 effectively inhibits PEDV replication in both Vero and LLC-PK1 cell lines. (**A**) LLC-PK1 cells were transfected with either ITGβ1 or an empty vector (1 µg). At 24 h post-transfection, cells were infected with PEDV at an MOI of 0.1 or mock-infected. Cells were harvested at 12, 16, and 20 hpi, and the expression of the PEDV N protein was analyzed by Western blotting, with GAPDH as a loading control. (**B and C**) The mRNA levels of the PEDV N protein and viral titers in the cell supernatants collected at 12, 16, and 20 hpi were quantified using qPCR and the TCID_50_ assay, respectively. (**D**) Vero cells were transfected with 1 µg of ITGβ1 or an empty vector, followed by PEDV infection (MOI = 0.1) at 24 h post-transfection. Cells were collected at 12, 16, and 20 hpi, and the expression level of the PEDV N protein was assessed by Western blotting. (**E and F**) Vero cells were transfected with 1 µg of ITGβ1 or an empty vector, and PEDV N protein expression and viral titers in supernatants harvested at specified time points were evaluated using qPCR and the TCID_50_ assay, respectively. (**G**) Western blotting was performed to evaluate the replication level of the PEDV N protein at 24 hpi in ITGβ1-overexpressing LLC-PK1^ITGβ1^ and Vero^ITGβ1^ cells infected with varying doses of PEDV, with wild-type LLC-PK1^WT^ and Vero^WT^ cells as controls. (**H**) ITGβ1-overexpressing LLC-PK1^ITGβ1^ and Vero^ITGβ1^ cells, along with their wild-type counterparts (LLC-PK1^WT^ and Vero^WT^), were infected with PEDV (MOI = 0.1). Cells were harvested at indicated time points, and PEDV replication was detected by Western blotting. Data are shown as means ± SD of three independent experiments. *, *P*  <  0.05; **, *P*  <  0.01; ns, not significant by two-tailed Student’s *t*-test (for two-group comparisons) or one-way ANOVA (for multi-group comparisons).

Previous research has reported that numerous pathogens exploit ITGβ1 to facilitate cellular infection. For instance, MV utilizes ITGβ1-mediated PI3K/Akt signaling to enhance its invasion of host cells (8). Similarly, the glycoprotein of rabies virus (RABV) interacts with ITGβ1, mediating its internalization and co-transport with RABV to late endosomes, thereby promoting RABV entry into peripheral cells (24). To assess the effect of ITGβ1 on PEDV replication, we overexpressed ITGβ1 in LLC-PK1 and Vero cells, followed by PEDV (0.1 MOI) infection 24 h post-transfection. Western blotting and qPCR showed that at 12, 16, and 20 hpi, PEDV N protein expression and viral mRNA copies were significantly lower in ITGβ1-overexpressing cells than in controls. (Fig. 3A, B, D and E). Virus titration assays, measured as the 50% tissue culture infective dose (TCID_50_), demonstrated that PEDV titers were reduced by 11.8-fold and 6.34-fold in ITGβ1-overexpressing groups, respectively (Fig. 3C and F). To further validate these findings, we utilized previously established ITGβ1-overexpressing cell lines (LLC-PK1^ITGβ1^ and Vero^ITGβ1^) and infected them with varying doses of PEDV. At 24 hpi, Western blotting was performed to detect viral protein expression. The results showed that, compared to wild-type cells, the expression of the PEDV N protein was significantly suppressed in both ITGβ1-overexpressing cells when infected with PEDV at MOI of 0.001, 0.005, 0.01, 0.1, and 1 (Fig. 3G). Similarly, when these ITGβ1-overexpressing cells were infected with 0.1 MOI of PEDV for 12, 24, and 36 hpi, the expression of the PEDV N protein was markedly reduced (Fig. 3H). These findings indicate that, in contrast to other viruses, overexpression of ITGβ1 does not promote viral proliferation but instead inhibits PEDV replication.

### LLC-PK1 cells with silenced ITGβ1 expression exhibit enhanced support for PEDV replication

**Fig 4:**
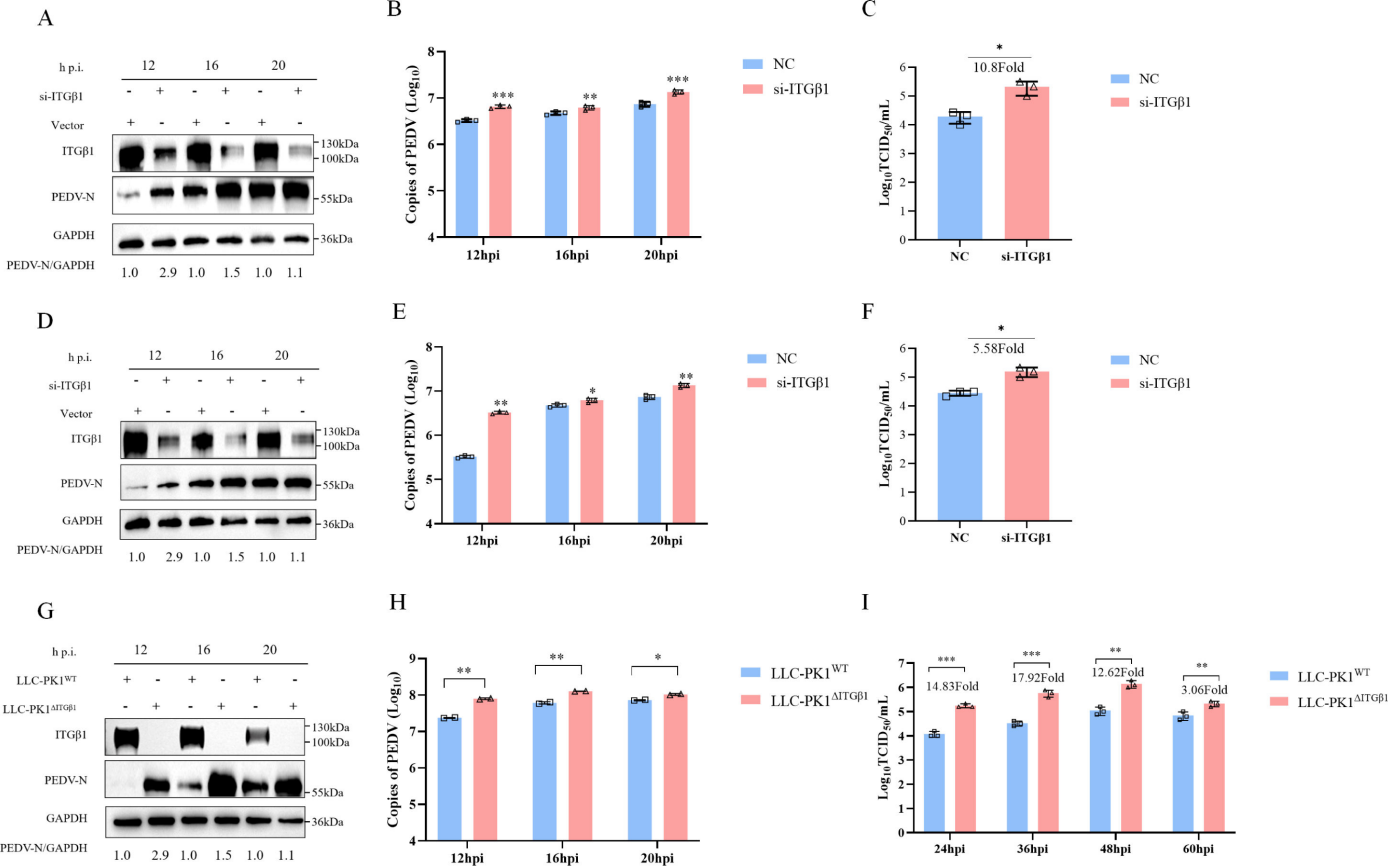
Suppression of endogenous ITGβ1 expression enhances PEDV replication. (**A**) LLC-PK1 cells were transfected with either a control siRNA (NC) or an ITGβ1-specific siRNA. At 36 h post-transfection, cells were infected with PEDV at an MOI of 0.1. Cell samples were harvested at 12, 16, and 20 hpi, and the expression of PEDV N protein was analyzed by Western blotting following protein extraction. (**B and C**) After 36 h of siRNA transfection, LLC-PK1 cells were infected with PEDV (MOI = 0.1). Supernatants were then collected at 12, 16, and 20 hpi, and the replication levels and viral titers of PEDV in ITGβ1-knockdown LLC-PK1 cells were determined by qPCR and TCID_50_ assay, respectively. (**D**) Similarly, the expression of PEDV N protein at 12, 16, and 20 hpi was evaluated by Western blotting in Vero cells with suppressed endogenous ITGβ1 expression. (**E and F**) The replication levels and virus titers of PEDV in ITGβ1-knockeddown Vero cells were measured by qPCR and TCID_50_ assay, respectively, at 12, 16, and 20 hpi. (**G and H**) LLC-PK1^WT^ and LLC-PK1^ΔITGβ1^ cells were infected with PEDV (MOI = 0.1), and cells and supernatants were collected at 12, 16, and 20 hpi. The levels of PEDV N protein and mRNA at the indicated time points were then assessed by Western blotting and qPCR, respectively. (**I**) Following infection with PEDV (MOI = 0.001), cell supernatants from LLC-PK1^WT^ and LLC-PK1^ΔITGβ1^ cells were collected at the specified time points, and PEDV titers were determined by TCID_50_ assay. Data are shown as means ± SD from three independent experiments. *, *P*  <  0.05; **, *P*  <  0.01; ***, *P*  <  0.001 by two-tailed Student’s *t*-test (for two-group comparisons) or one-way ANOVA (for multi-group comparisons).

Given the inhibitory effect of ITGβ1 overexpression on PEDV replication, we utilized siRNA to knock down the expression of endogenous ITGβ1 in LLC-PK1 and Vero cells to assess its impact on PEDV replication. Through Western blotting, qPCR, and virus titer detection, it was observed that downregulation of ITGβ1 expression via siITGβ1 in LLC-PK1 and Vero cells significantly augmented PEDV proliferation at 12, 16, and 20 hpi (Fig. 4A, B, D and E). Analysis of progeny virus titers revealed that, following the knockdown of endogenous ITGβ1 protein, PEDV titers increased by 10.8-fold and 5.58-fold, respectively, compared to the control group (Fig. 4C and F). To further corroborate these results, we employed the ITGβ1 knockout cell line LLC-PK1^ΔITGβ1^, which had been previously established in our laboratory. The data indicated that, compared to wild-type cells, LLC-PK1^ΔITGβ1^ cells deficient in ITGβ1 were more permissive to PEDV infection, exhibiting significantly increased levels of viral N protein and mRNA (Fig. 4G and H). Moreover, the titer of progeny virus was also substantially increased, reaching up to 17.92-fold higher than that of the control (Fig. 4I). These results collectively suggest that the suppression or deletion of endogenous ITGβ1 expression in cells not only negates its inhibitory effect but also promotes PEDV replication, indicating that ITGβ1 may function as a potential host restriction factor against PEDV.

### ITGβ1 is essential for mediating an effective antiviral response during PEDV infection

**Fig 5:**
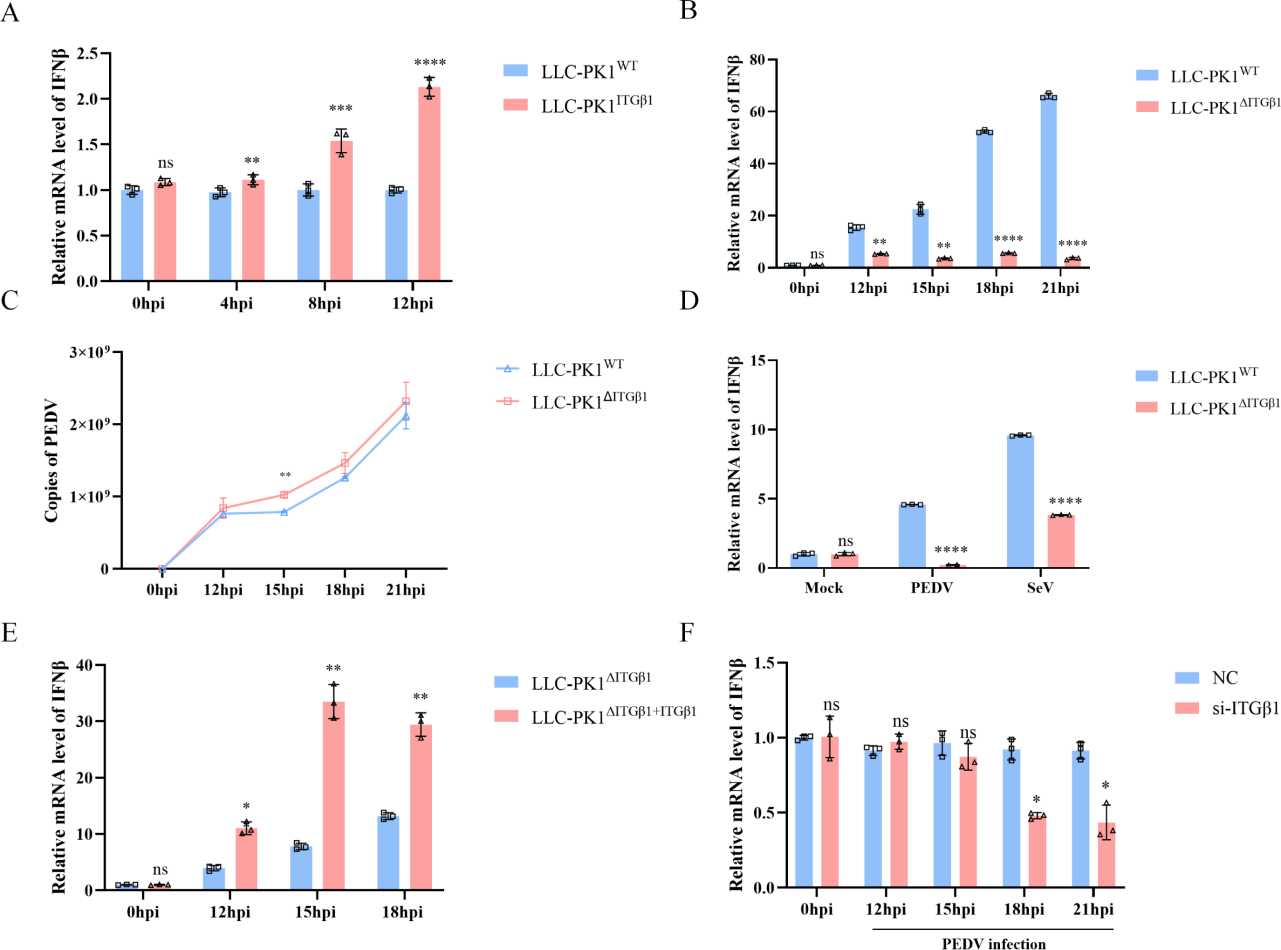
ITGβ1 is essential for the PEDV-induced antiviral response. (**A**) LLC-PK1^WT^ and LLC-PK1^ΔITGβ1^ cells were infected with PEDV at an MOI of 0.1. Changes in IFN-β mRNA levels were measured by qPCR at 0, 4, 8, and 12 hpi. (**B**) LLC-PK1^WT^ and LLC-PK1^ΔITGβ1^ cells were infected with PEDV (MOI = 0.1), and IFN-β mRNA levels were analyzed by qPCR at 12, 15, 18, and 21 hpi. (**C**) LLC-PK1^WT^ and LLC-PK1^ΔITGβ1^ cells were infected with PEDV at an MOI of 0.1, and cells were collected at 12, 15, 18, and 21 hpi. The cell supernatants were then analyzed by qPCR to determine the IFN-β mRNA levels, as well as the copy number of the PEDV-N protein. (**D**) LLC-PK1^WT^ and LLC-PK1^ΔITGβ1^ cells were inoculated with PEDV (MOI = 0.1) and SeV at 80HAU/mL, respectively. Cells were collected at 18 hpi, and changes in IFN-β mRNA levels were detected by qPCR. (**E**) LLC-PK1^ΔITGβ1^ cells were transfected with ITGβ1 or an empty vector, followed by infection with PEDV (MOI = 0.1). At 18 hpi, cells were collected, and IFN-β mRNA levels were analyzed by qPCR. (**F**) LLC-PK1 cells were transfected with negative control (NC) and siRNA, followed by infection with PEDV (MOI = 0.1). Samples were collected at specified time points, and changes in IFN-β mRNA levels were measured by qPCR. Data are shown as means ± SD of three independent experiments. *, *P*  <  0.05; **, *P*  <  0.01; ***, *P*  <  0.001; ****, *P* < 0.0001; ns, not significant by one-way ANOVA.

To elucidate the role of ITGβ1 in the host antiviral response, we conducted a series of experiments. First, wild-type LLC-PK1^WT^ cells and ITGβ1-overexpressing LLC-PK1^ITGβ1^ cells were infected with PEDV at an MOI of 0.1, and changes in IFN-β mRNA levels were assessed by qPCR. We found that at 4, 8, and 12 hpi, IFN-β mRNA levels were significantly higher in LLC-PK1^ITGβ1^ cells compared to LLC-PK1^WT^ cells (Fig. 5A). To further investigate the importance of ITGβ1 in PEDV-induced antiviral response, we performed a loss-of-function experiment using the ITGβ1 knockout cell line LLC-PK1^ΔITGβ1^. In contrast to the previous results, LLC-PK1^ΔITGβ1^ cells exhibited only low levels of IFN-β mRNA, suggesting that ITGβ1 plays a critical role in inducing IFN-β expression during PEDV infection (Fig. 5B). Viral copy number analysis further revealed that LLC-PK1^ΔITGβ1^ cells harbored a higher viral load than LLC-PK1^WT^ cells, with a significant difference observed at 15 hpi (Fig. 5C). Previous studies have shown that Sendai virus (SeV) can induce early IFN-β expression but suppress it at later stages by degrading interferon regulatory factor 3 (IRF3) after 12 hpi (25). To explore this function, we infected LLC-PK1^WT^ and LLC-PK1^ΔITGβ1^ cells with SeV or PEDV and measured IFN-β expression at 18 hpi. The results demonstrated that the IFN-β expression was significantly reduced in LLC-PK1^ΔITGβ1^ cells infected with either SeV or PEDV compared to LLC-PK1^WT^ cells, indicating that the absence of ITGβ1 impairs the IFN-β production pathway (Fig. 5D). To further confirm these findings, we complemented ITGβ1 in LLC-PK1^ΔITGβ1^ cells and infected them with PEDV at an MOI of 0.1. This led to a rapid increase in IFN-β mRNA levels, further reinforcing the role of ITGβ1 in inducing IFN-β production (Fig. 5E). Additionally, to validate the observations in LLC-PK1^ΔITGβ1^ cells, we downregulated the endogenous expression of ITGβ1 in LLC-PK1^WT^ cells using siRNA. Following 18 h of PEDV infection at an MOI of 0.1, IFN-β mRNA levels were significantly downregulated (Fig. 5F), indicating that reduced ITGβ1 expression inhibits IFN-β production. Taken together, these findings demonstrate that ITGβ1 is indispensable for the antiviral response induced by PEDV infection.

### ITGβ1 enhances SeV-induced IFN-β production

**Fig 6:**
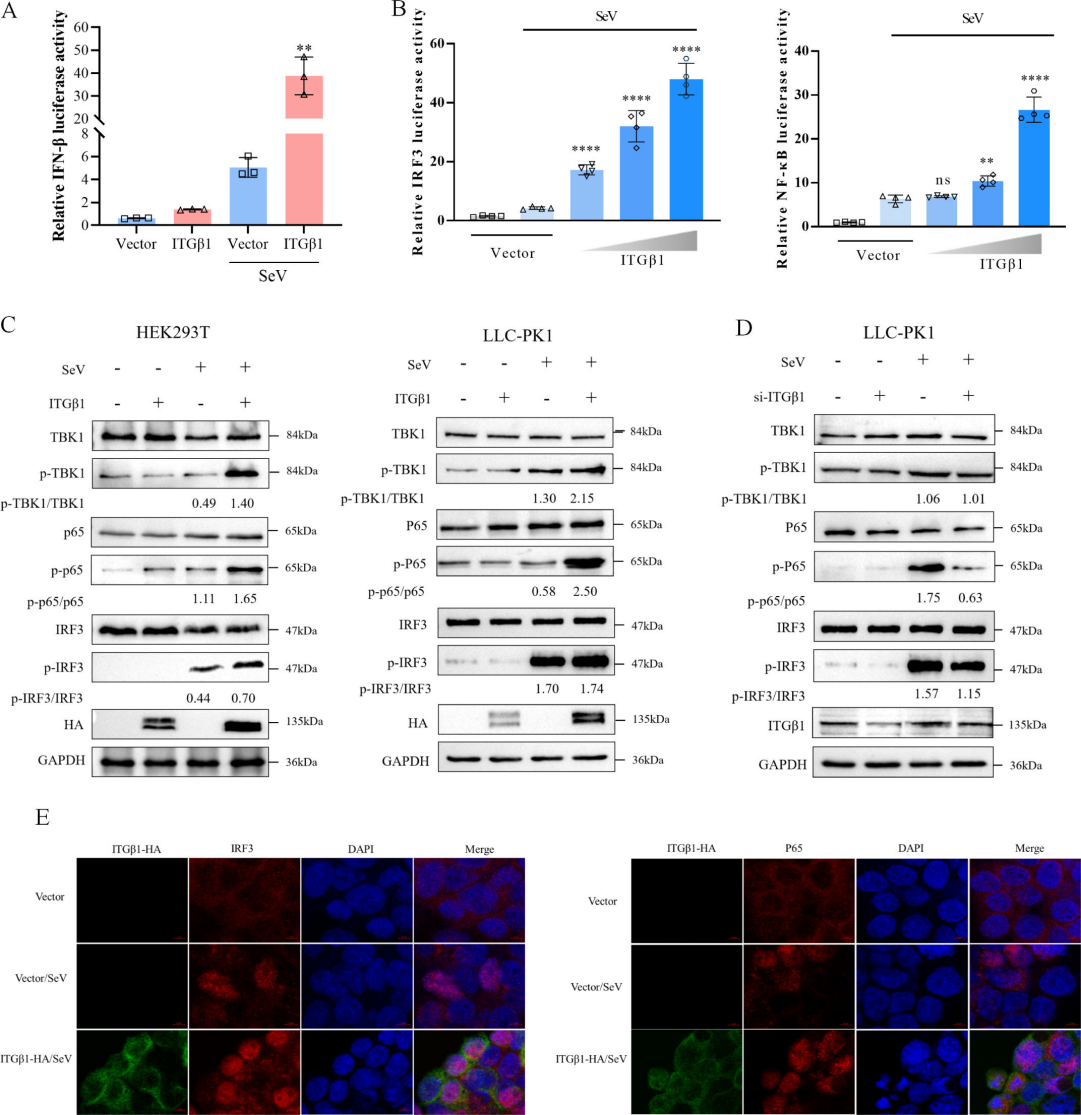
ITGβ1 promotes SeV-mediated IFN-β production. (**A**) HEK293T cells were co-transfected with ITGβ1 or an empty vector, along with the IFN-β-Luc reporter plasmid and the pRL-TK plasmid. At 24 h post-transfection, cells were either mock-treated or treated with SeV for 12 h. Subsequently, the cells were harvested, and the IFN-β promoter activity was assessed using a dual luciferase assay. (**B**) Cells were co-transfected with ITGβ1 or an empty vector, together with either the IRF3-Luc or NF-κB-Luc reporter plasmid and the pRL-TK plasmid. After SeV stimulation, cells were collected at 12 h post-treatment and analyzed using a dual luciferase assay. (**C**) HEK 293T cells or LLC-PK1 cells were transfected with ITGβ1 or an empty vector (1 µg each). At 24 hours post-transfection, cells were treated with SeV or left untreated for 12 hours. Subsequently, the cell lysates were analyzed by immunoblotting using antibodies specific for phosphorylated IRF3 or p65, as well as total IRF3 or p65. (**D**) LLC-PK1 cells were transfected with si-ITGβ1 or NC. At 24 h post-transfection, the cells were either treated with Sendai virus (SeV) or left untreated for 12 h. Cell lysates were then collected and subjected to Western blotting analysis using antibodies against phosphorylated TBK1 (p-TBK1), IRF3 (p-IRF3) or phosphorylated p65 (P-P65), as well as antibodies against total TBK1, IRF3 or total p65. (**E**) HEK293T cells were transfected with ITGβ1 or an empty vector. At 24 h after transfection, cells were either mock-infected or infected with SeV for 12 h, as described previously. Cells were fixed, permeabilized, and incubated with primary antibodies: rabbit anti-IRF3 or anti-p65, and mouse anti-ITGβ1. Cells were then incubated with goat anti-rabbit 594-conjugated secondary antibody (labeled in red) and goat anti-mouse 488-conjugated secondary antibody (labeled in green). Nuclei were stained with DAPI, and images were acquired using a laser scanning confocal microscope. Data are shown as means ± SD of three independent experiments. **, *P*  <  0.01; ****, *P* < 0.0001; ns, not significant by one-way ANOVA.

SeV is widely recognized for its ability to induce IFN-β production. To determine the effect of ITGβ1 on IFN-β production, HEK293T cells were co-transfected with an ITGβ1 eukaryotic expression vector, along with IFN-β-Luc and pRL-TK plasmids. Following SeV treatment, we found that the ITGβ1 expression significantly increased the activity of the IFN-β promoter (Fig. 6A). Since the induction of IFN-I requires the activation of transcription factors such as IRF3 and NF-κB, we employed dual-luciferase reporter assays to evaluate the effect of ITGβ1 expression on IRF3-Luc and NF-κB-Luc reporter activities. The results demonstrated that ITGβ1 enhanced SeV-induced IRF3 and NF-κB promoter activity in a dose-dependent manner (Fig. 6B). Additionally, we further examined the effect of ITGβ1 expression on SeV-induced phosphorylation levels of TBK1, IRF3, and NF-κB. The results showed that overexpression of ITGβ1 in both HEK293T and LLC-PK1 cells significantly enhanced SeV-induced phosphorylation of TBK1, IRF3, and NF-κB (Fig. 6C). In contrast, when ITGβ1 was knocked down in LLC-PK1 cells, the phosphorylation levels of TBK1, IRF3, and NF-κB were significantly decreased (Fig. 6D). Concurrently, ITGβ1 overexpression facilitated nuclear translocation of IRF3 and NF-κB (Fig. 6E). These findings collectively indicate that ITGβ1 enhances SeV-induced IFN-β promoter activity, which is associated with the activation of both IRF3 and NF-κB signaling pathways. Thus, ITGβ1 plays a positive regulatory role in the IFN-I signaling pathway.

### ITGβ1 enhances MDA5-mediated IFN-β production

**Fig 7:**
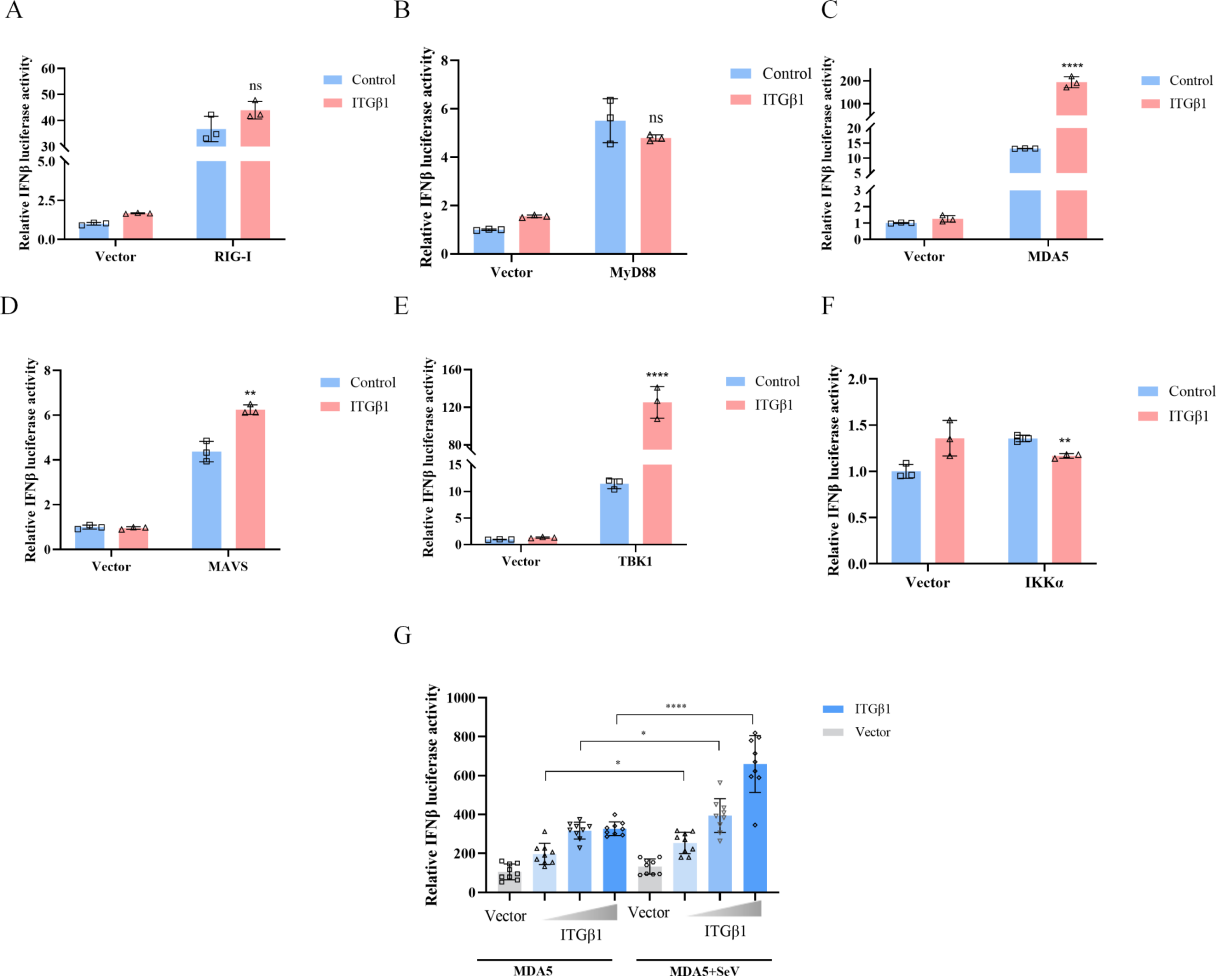
ITGβ1 promotes MDA5-mediated IFN-β production. HEK293T cells were co-transfected with plasmids encoding IFN-β-Luc, pRL-TK (as a control for transfection efficiency), and pcDNA3.1-ITGβ1-HA, along with plasmids expressing RIG-I (**A**), MyD88 (**B**), MDA5 (**C**), MAVS (**D**), TBK1 (**E**), and IKKα (**F**) using plasmid amounts of 0.5, 0.25, and 0.05 µg, respectively. Renilla luciferase activity was used as a normalization control. At 24 h post-transfection, cell lysates were collected, and the activities of each molecule were assessed using a dual luciferase reporter assay. In a separate experiment (**G**), HEK293T cells were transfected with plasmids encoding ITGβ1 (0.5, 1, and 1.5 µg), MDA5 (0.25 µg), or an empty vector, along with pRL-TK (0.05 µg). At 24 h post-transfection, cells were either treated with SeV for 12 h or left untreated. Cell lysates were then collected, and luciferase activity was analyzed using a dual-luciferase reporter assay. Data are shown as means ± SD of least three independent experiments. *, *P*  <  0.05; **, *P*  <  0.01; ****, *P* < 0.0001; ns, not significant by two-tailed Student’s *t*-test (for two-group comparisons) or one-way ANOVA (for multi-group comparisons).

To elucidate the regulatory mechanism of ITGβ1 in IFN-I production, we examined its effects on IFN-I promoter activity mediated by various upstream signaling molecules in the IFN-I pathway. Luciferase reporter assays demonstrated that the presence or absence of ITGβ1 did not significantly affect IFN-β promoter activity induced by RIG-I and MyD88 (Fig. 7A and B). However, the presence of ITGβ1 markedly enhanced IFN-β promoter activity triggered by MDA5 and its downstream signaling components, including MAVS, TBK1, and IKKα, indicating that ITGβ1 primarily promotes IFN-β production by targeting MDA5 (Fig. 7C through F). Notably, ITGβ1 overexpression dose-dependently increased MDA5-mediated IFN-I promoter activity and significantly amplified IFN-I promoter activity induced by SeV and MDA5, compared to the control (Fig. 7G). These results demonstrated that ITGβ1 acts as a critical activator of the MDA5-dependent antiviral response (Fig. 7G).

### ITGβ1 enhances dsRNA-induced IFN-β production

**Fig 8:**
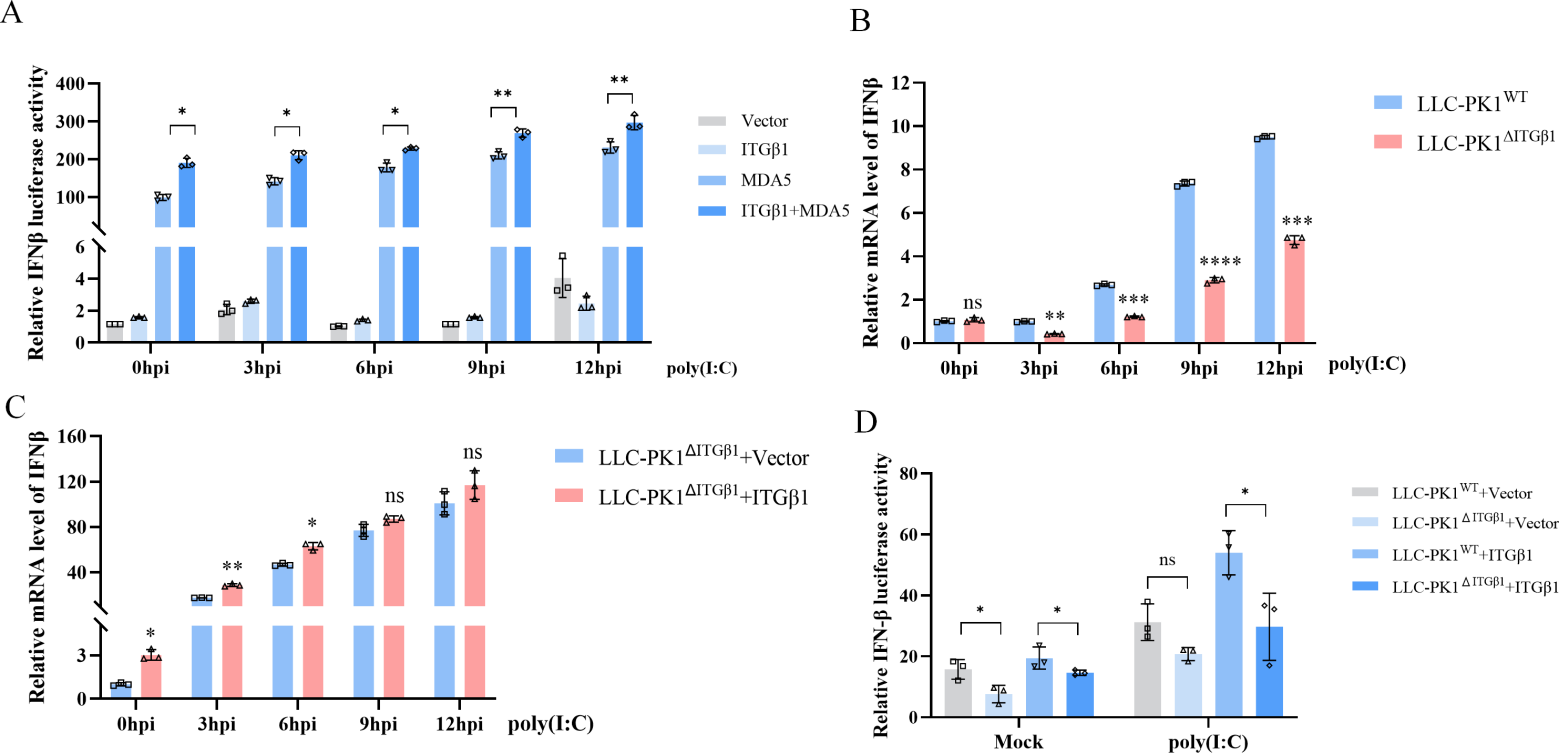
ITGβ1 enhances dsRNA-induced IFN-β production. (**A**) HEK293T cells were co-transfected with ITGβ1, MDA5 (or an empty vector serving as a control), the IFN-β-Luc reporter plasmid, and the pRL-TK plasmid using plasmid amounts of 0.5, 0.5, 0.25, and 0.05 µg, respectively. At 24 h post-transfection, cells were either left untreated or stimulated with poly(I:C). Cell lysates were collected at 0, 3, 6, 9, and 12 h post-stimulation, and IFN-β levels were quantified using a dual-luciferase reporter assay. (**B**) Wild-type LLC-PK1^WT^ cells and ITGβ1-knockout LLC-PK1^ΔITGβ1^ cells stimulated with poly(I:C). Cells were harvested at 0, 3, 6, 9, and 12 h post-stimulation, and changes in IFN-β mRNA levels were analyzed by qPCR. (**C**) LLC-PK1^ΔITGβ1^ cells were co-transfected with ITGβ1 or an empty vector. At 24 h post-transfection, cells were either stimulated with poly(I:C) or left unstimulated. Cells were collected at 0, 3, 6, 9, and 12 h post-stimulation, and IFN-β mRNA expression was measured by qPCR. (**D**) LLC-PK1^WT^ and LLC-PK1^ΔITGβ1^ cells were co-transfected with ITGβ1, MDA5 (or an empty vector), the IFN-β-Luc reporter plasmid, and the pRL-TK plasmid at transfection ratios of 0.5, 0.5, 0.25, and 0.05 µg, respectively. At 24 h post-transfection, cells were treated with poly(I:C) for 6 h, and IFN-β activity was assessed using a dual-luciferase reporter assay. Data are presented as means ± SD from three independent experiments. *, *P* < 0.05; **, *P* < 0.01; ***, *P* < 0.001; ****, *P* < 0.0001; ns, not significant by two-tailed Student’s *t*-test (for two-group comparisons) or one-way ANOVA (for multi-group comparisons).

MDA5 is capable of recognizing long double-stranded RNA (dsRNA), and high molecular weight poly(I:C) is a direct activator of MDA5 (26). To investigate the role of ITGβ1 in this process, HEK293T cells were co-transfected with plasmids encoding ITGβ1, MDA5, and an IFN-β luciferase reporter (IFN-β-luc), followed by stimulation with poly(I:C). The results showed that MDA5 expression alone significantly enhanced IFN-β promoter activation. Although transfection of ITGβ1 alone exerted a negligible influence on IFN-β activation, the combination of ITGβ1 and MDA5 markedly potentiated IFN-β promoter activity following poly(I:C) stimulation (Fig. 8A). To evaluate the impact of ITGβ1 deficiency on IFN production, we used LLC-PK1^ΔITGβ1^. The absence of ITGβ1 significantly suppressed poly(I:C)-induced IFN-β mRNA expression (Fig. 8B). However, when ITGβ1 was reintroduced into LLC-PK1^ΔITGβ1^ cells through overexpression, the stimulatory effect of poly(I:C) on the IFN-β promoter was restored (Fig. 8C). To further corroborate the importance of ITGβ1 in dsRNA-induced IFN-β production, we assessed IFN-β promoter activity in LLC-PK1^ΔITGβ1^ cells. The results demonstrated that the absence of endogenous ITGβ1 suppressed IFN-β promoter activation (Fig. 8D), indicating that ITGβ1 can enhance dsRNA-induced IFN-β production.

### ITGβ1 interacts with MDA5 and promotes MDA5 oligomerization

**Fig 9:**
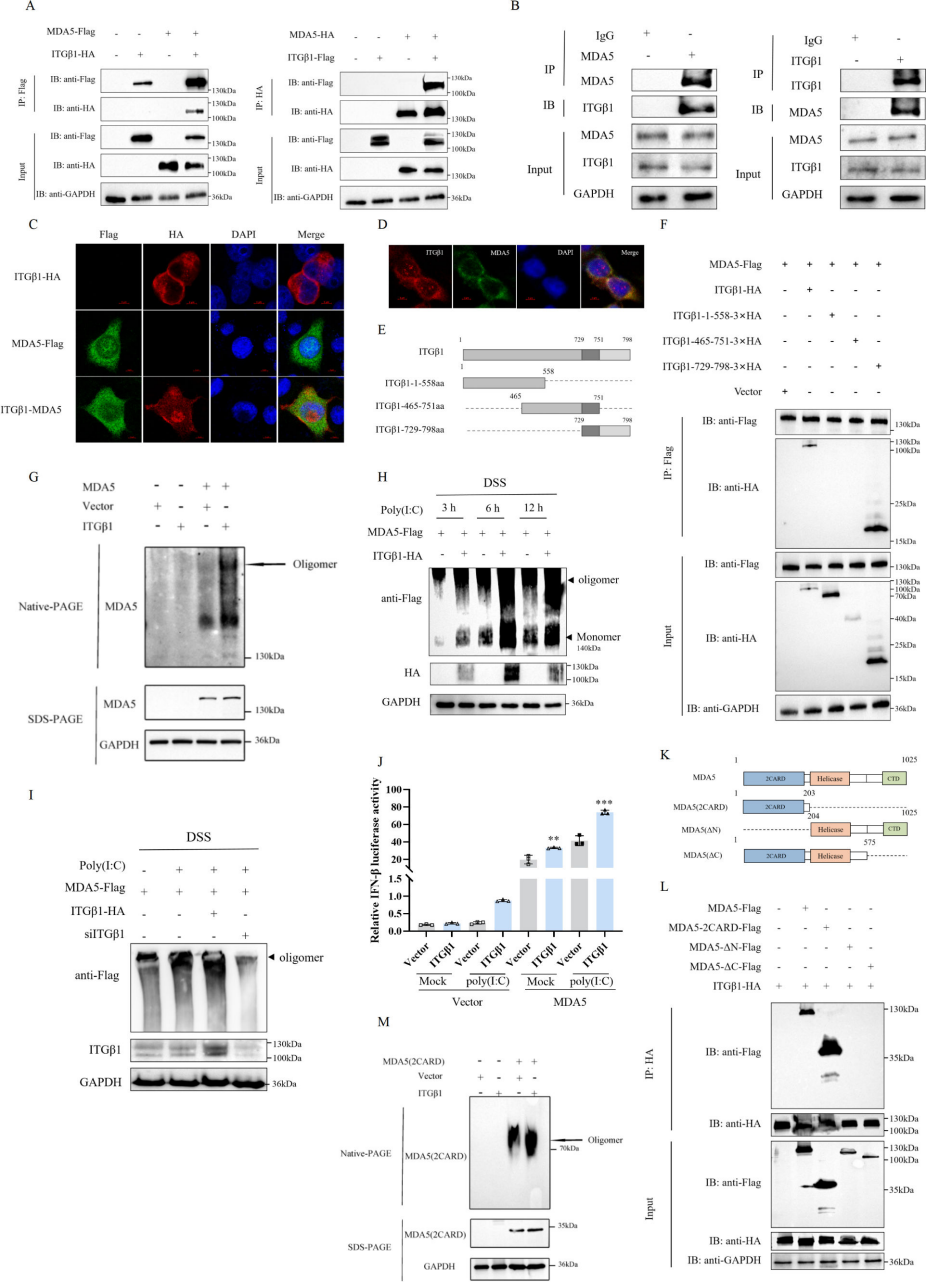
ITGβ1 interacts with MDA5 and promotes MDA5 oligomerization. (**A**) HEK293T cells were transfected with an empty vector, HA-tagged ITGβ1 or MDA5, and Flag-tagged ITGβ1 or MDA5, either individually or in combination. At 24 h post-transfection, cells were harvested, and co-immunoprecipitation (Co-IP) analysis was performed using anti-Flag or anti-HA magnetic beads. (**B**) After HEK 293T cells reached confluency (formed a monolayer), the cells were harvested. Magnetic beads were incubated with either anti-MDA5 antibody or anti-ITGβ1 antibody, followed by Co-IP analysis. (**C**) HEK293T cells were transfected with an empty vector, pcDNA3.1-ITGβ1-HA, and pcDNA3.1-MDA5-Flag. After 24 h, the cells were permeabilized, fixed, and stained with rabbit anti-HA (red) and mouse anti-Flag (green) primary antibodies. Cells were then incubated with goat anti-rabbit 594-conjugated (red) and goat anti-mouse 488-conjugated (green) secondary antibodies, followed by DAPI staining. Images were visualized using laser confocal microscopy. (**D**) HEK 293T cells were fixed and permeabilized, then incubated with primary antibodies: mouse anti-MDA5 antibody (green) and rabbit anti-ITGβ1 antibody (red). Subsequent incubation was performed with secondary antibodies: goat anti-rabbit IgG conjugated to Alexa Fluor 594 (red) and goat anti-mouse IgG conjugated to Alexa Fluor 488 (green). After DAPI staining, the cells were observed under a laser confocal microscope. (**E**) The structural domains of ITGβ1 were predicted using the SMART online analysis tool (http://smart.embl-heidelberg.de/). Truncated constructs were generated based on the results, with the dark gray region representing the transmembrane domain, flanked by the extracellular domain and cytoplasmic tail, respectively. (**F**) HEK293T cells were co-transfected with pCDNA3.1-MDA5-Flag and either an empty vector or full-length/truncated ITGβ1 plasmids. At 24 h post-transfection, cells were lysed, and Co-IP was performed using anti-Flag antibody-conjugated magnetic beads, followed by protein immunoblotting analysis. (**G**) HEK293T cells were transfected or co-transfected with an empty vector, HA-tagged ITGβ1, and Flag-tagged MDA5. After 24 h, native PAGE and denaturing PAGE were employed for Western blotting analysis to detect exogenous MDA5 oligomerization using a Flag antibody. (**H**) LLC-PK1 cells were transfected or co-transfected with empty vector, HA-tagged ITGβ1, Flag-tagged MDA5, or si-ITGβ1, respectively. At 24 h post-transfection, the cells were stimulated with poly(I:C) (2 µg/mL). Cell samples were collected at 3, 6, and 12 h post-stimulation, subjected to disuccinimidyl suberate (DSS) cross-linking, and then analyzed for MDA5 oligomerization by SDS-PAGE and Western blotting. (**I**) HEK 293T cells were transfected or co-transfected with empty vector, HA-tagged ITGβ1, or Flag-tagged MDA5, respectively. At 24 h post-transfection, the cells were stimulated with poly(I:C) (2 µg/mL) for 12 h. After stimulation, cell samples were collected, treated with DSS cross-linking, and MDA5 oligomerization was detected by SDS-PAGE and Western blotting. (**J**) HEK293T cells were transfected with an empty vector, ITGβ1, MDA5, IFN-β-Luc plasmid, and pRL-TK plasmid at ratios of 0.5, 0.5, 0.25, and 0.05 µg, respectively. At 24 h post-transfection, cells were stimulated with poly(I:C) or left unstimulated. After 12 h, cells were collected, and IFN-β activation was measured using a dual luciferase reporter system. (**K**) A schematic diagram illustrating the truncation of MDA5 is provided. (**L**) HEK293T cells were co-transfected with an empty vector, HA-tagged ITGβ1, and Flag-tagged full-length MDA5 or various truncated MDA5 fragments. At 24 h post-transfection, Co-IP was performed using anti-HA magnetic beads, followed by Western blotting analysis. (**M**) HEK293T cells were co-transfected with pcDNA3.1, pcDNA3.1-ITGβ1-HA, and pcDNA3.1-MDA5 (2CARD)-Flag at ratios of 0.5, 0.5, and 0.5 µg, respectively. At 24 h post-transfection, cell lysates were collected, and MDA5 oligomerization was detected by native PAGE and denaturing PAGE using immunoblotting techniques. Data are presented as means ± SD from three independent experiments. **, *P* < 0.01; ***, *P* < 0.001 by one-way ANOVA.

Previous studies have identified ITGβ1 as a critical activator of MDA5. To determine whether ITGβ1 and MDA5 physically interact, we overexpressed both proteins in HEK293T cells and performed co-immunoprecipitation (Co-IP) using anti-Flag or anti-HA magnetic beads. The Co-IP results demonstrated a significant interaction between ITGβ1 and MDA5 (Fig. 9A). We further examined the endogenous interaction between ITGβ1 and MDA5 in cells using Co-IP assays. The results showed that the interaction between MDA5 and ITGβ1 was also detected in HEK293T cells (Fig. 9B). ITGβ1 is a known type I transmembrane protein (2). In previous experiments, we investigated the subcellular localization of ITGβ1 in HEK293T cells through permeabilized and non-permeabilized treatments. The results demonstrated that ITGβ1 expression was mainly localized to the cell membrane under non-permeabilized conditions; after permeabilization with Triton X-100, ITGβ1 expression was also observed in the cytoplasm (Fig. S1). This finding is consistent with results reported in the literature (27). On this basis, we observed the subcellular co-localization of ITGβ1 and MDA5 using confocal microscopy. The results further confirmed that the co-localization of ITGβ1 and MDA5 in cells could be detected both under ITGβ1 overexpression and endogenous expression conditions (Fig. 9C and D). ITGβ1 consists of three functional domains: extracellular matrix adhesion (1–729 aa), cell transport modulation (730–751 aa), and bidirectional signal transduction processes (752–798 aa) (2). To further investigate the specific domain of ITGβ1 responsible for its interaction with MDA5, we generated truncated versions of ITGβ1 (Fig. 9E) and performed Co-IP with MDA5, respectively. The results revealed that ITGβ1 729–798 aa, which includes the cytoplasmic tail, interacted with MDA5, whereas ITGβ1 465–751 aa did not (Fig. 9F), indicating that the cytoplasmic tail of ITGβ1 mediates its interaction with MDA5. Notably, MDA5 oligomerization is essential for MDA5-dependent antiviral signaling (28, 29). In order to ascertain whether ITGβ1 could induce the oligomerization of MDA5, we assessed the oligomerization status of the MDA5 protein in ITGβ1-overexpression cells using native-PAGE electrophoresis. The results showed that ITGβ1 expression markedly enhanced MDA5 oligomerization compared to the control (Fig. 9G).

Furthermore, we overexpressed ITGβ1 in HEK293T cells. At 24 h post-transfection, the cells were stimulated with poly(I:C) to mimic viral RNA-induced MDA5 oligomerization. At 3 h, 6 h, and 12 h after poly(I:C) stimulation, the oligomerization status of endogenous MDA5 was investigated using disuccinimidyl suberate (DSS) cross-linking technology. The results showed that ITGβ1 overexpression significantly enhanced poly(I:C)-induced MDA5 oligomer formation; this effect was detectable as early as 3 h and persisted for at least 12 h. Notably, both MDA5 oligomerization and ITGβ1 protein levels reached their peaks at 6 h post-stimulation (Fig. 9H). To confirm the specificity of ITGβ1’s function, we knocked down ITGβ1 expression in LLC-PK1 cells using small interfering RNA (siRNA), followed by DSS cross-linking analysis. The results demonstrated that compared with wild-type cells, ITGβ1 knockdown significantly reduced poly(I:C)-induced MDA5 oligomer formation. Similarly, overexpression of ITGβ1 notably promoted MDA5 oligomerization (Fig. 9I), which further supports that ITGβ1 exerts a positive regulatory role in MDA5 activation. To evaluate whether the ITGβ1-induced MDA5 oligomerization enhances IFN-β production, we transfected HEK293T cells with ITGβ1 or an empty vector and measured luciferase activity. We found that ITGβ1 expression augmented the activation of the IFN-β promoter mediated by MDA5 upon poly(I:C) stimulation (Fig. 9J).

MDA5 contains two N-terminal CARD domains, a central helicase domain, and a C-terminal CTD domain (30). To elucidate the molecular mechanisms underlying ITGβ1-mediated oligomerization of MDA5, we utilized eukaryotic expression vectors encoding the key functional domains of MDA5: 2CARD (1–203 aa), MDA5-ΔN (204–1,025 aa), and MDA5-ΔC (1–575 aa), and performed Co-IP experiments with ITGβ1. The Co-IP results demonstrated that ITGβ1 specifically interacted with and precipitated the MDA5-2CARD domains (Fig. 9K and L), indicating that the primary interaction site between ITGβ1 and MDA5 resides within the 2CARD domains. Furthermore, to further investigate the role of ITGβ1 in MDA5 oligomerization, we assessed the oligomerization of MDA5-2CARD in the presence of ITGβ1. The results revealed that ITGβ1 significantly promoted the oligomerization of MDA5-2CARD (Fig. 9M), suggesting that ITGβ1 facilitates MDA5 activation by enhancing the oligomerization of the 2CARD domains. In summary, these findings demonstrate that ITGβ1 can promote MDA5 oligomerization and enhance its ability to recruit viral RNA, thereby mediating the production of downstream IFN-β.

## DISCUSSION

PEDV infection poses a severe threat to the global pig farming industry, primarily causing diarrhea and high mortality rates in newborn piglets. Viral infection begins with the attachment of the virus to host cell surface. ITGβ1, a cell adhesion molecule localized on the cell membrane, is ubiquitous in various cell types and plays a pivotal role in receptor-mediated viral endocytosis and cellular entry for numerous viruses, including reovirus, RABV, vaccinia virus, cytomegalovirus, metapneumovirus, and SARS-CoV-2 (8, 11, 24, 31–35). In addition to its role in viral entry, integrins are essential signal transduction proteins that mediate interactions between cells and extracellular matrix proteins or other cells via cell surface ligands. They are also involved in complex immune regulatory signaling pathways (36–38). However, many biological functions of integrin in signal transduction remain elusive, making the study of integrin-mediated signaling a highly active and important area of research.

In our study, we found that PEDV infection significantly upregulates ITGβ1 expression in host cells. Additionally, we observed that during PEDV infection, host cells enhance the transcription of ITGβ1 by upregulating the transcriptional regulator c-Myc and recruiting it to the promoter region of ITGβ1. c-Myc, a multifunctional transcription factor, has been demonstrated to bind to the core promoter regions of most transcriptionally active genes, thereby enhancing their transcriptional elongation (39, 40). Our findings indicated that the active transcription of the ITGβ1 gene in PEDV-infected host cells was primarily associated with the activation of its promoter activity mediated by c-Myc regulation. Based on these observations, we hypothesize that the abundantly expressed host protein ITGβ1 may play a role in PEDV infection. To evaluate the potential function of ITGβ1 during PEDV infection, we conducted further investigations. Contrary to other viruses such as vaccinia virus and RABV, where overexpression of ITGβ1 enhances viral replication (8, 24), we observed that increased ITGβ1 expression significantly suppressed PEDV replication *in vitro in* passaged cell lines. Conversely, knockdown or loss of endogenous ITGβ1 expression led to a 17.92-fold increase in PEDV progeny virus titer, accompanied by a marked enhancement in viral proliferation. These results suggest that ITGβ1 might function as a potential antiviral host factor during PEDV infection. This observation was further corroborated in the ITGβ1-deficient LLC-PK1^ΔITGβ1^ cell line. Upon PEDV infection, LLC-PK1^ΔITGβ1^ cells exhibited impaired production of IFN-I and an increased PEDV gene copy number, indicating that the loss of ITGβ1 may disrupt the IFN-I signaling pathway. Remarkably, re-expression of ITGβ1 in the ITGβ1-deficient LLC-PK1^ΔITGβ1^ cells restored the loss of IFN-I, with IFN-I mRNA levels rapidly returning to normal. These findings clearly demonstrate that ITGβ1 likely plays a crucial role in the innate immune response against PEDV, and its antiviral effect is closely associated with PEDV infection.

Viral antagonism of the host innate immune response is critical for viral replication and often influences the progression of viral infections. IFN-I constitutes a pivotal component of the innate immune response, with their gene expression regulated by transcription factors such as IRF3 and NF-κB (41). In this study, we demonstrated that ITGβ1 overexpression significantly enhanced SeV-mediated induction of IFN-β. Concurrently, ITGβ1 exhibited a stimulatory effect on the activation and nuclear translocation of IRF3 and NF-κB. Following ITGβ1 stimulation, increased phosphorylation levels of IRF3 and NF-κB facilitated their nuclear translocation and binding to the IFN-β promoter, thereby augmenting its expression. Furthermore, ITGβ1 synergized with MDA5, MAVS, TBK1, and IKKα to enhance IFN-β transcription in HEK293T cells, while exerting no discernible impact on the RIG-I-mediated innate antiviral response. These findings indicate that ITGβ1 positively modulates the MDA5-MAVS-IFN-β pathway in an IFN-I-mediated innate immune response through a RIG-I-independent, MDA5-dependent mechanism. These observations underscore the potential role of ITGβ1 in safeguarding the host against viral challenges. Notably, prior studies have identified ITGβ1 as a receptor or co-receptor facilitating infection by multiple viruses, including HCMV), EboV, Kaposi’s sarcoma-associated herpes virus, and Epstein-Barr virus (9, 10, 42, 43). A recent study further reported that ITGβ1 interacts with the RABV glycoprotein, serving as a critical host factor for early RABV entry (24). In contrast, our findings reveal an opposing role of ITGβ1 in PEDV infection compared to these earlier reports. We hypothesize that as a multifunctional protein, ITGβ1 may exert divergent effects depending on the specific viral pathogen, highlighting its context-dependent role in viral pathogenesis.

**Fig 10:**
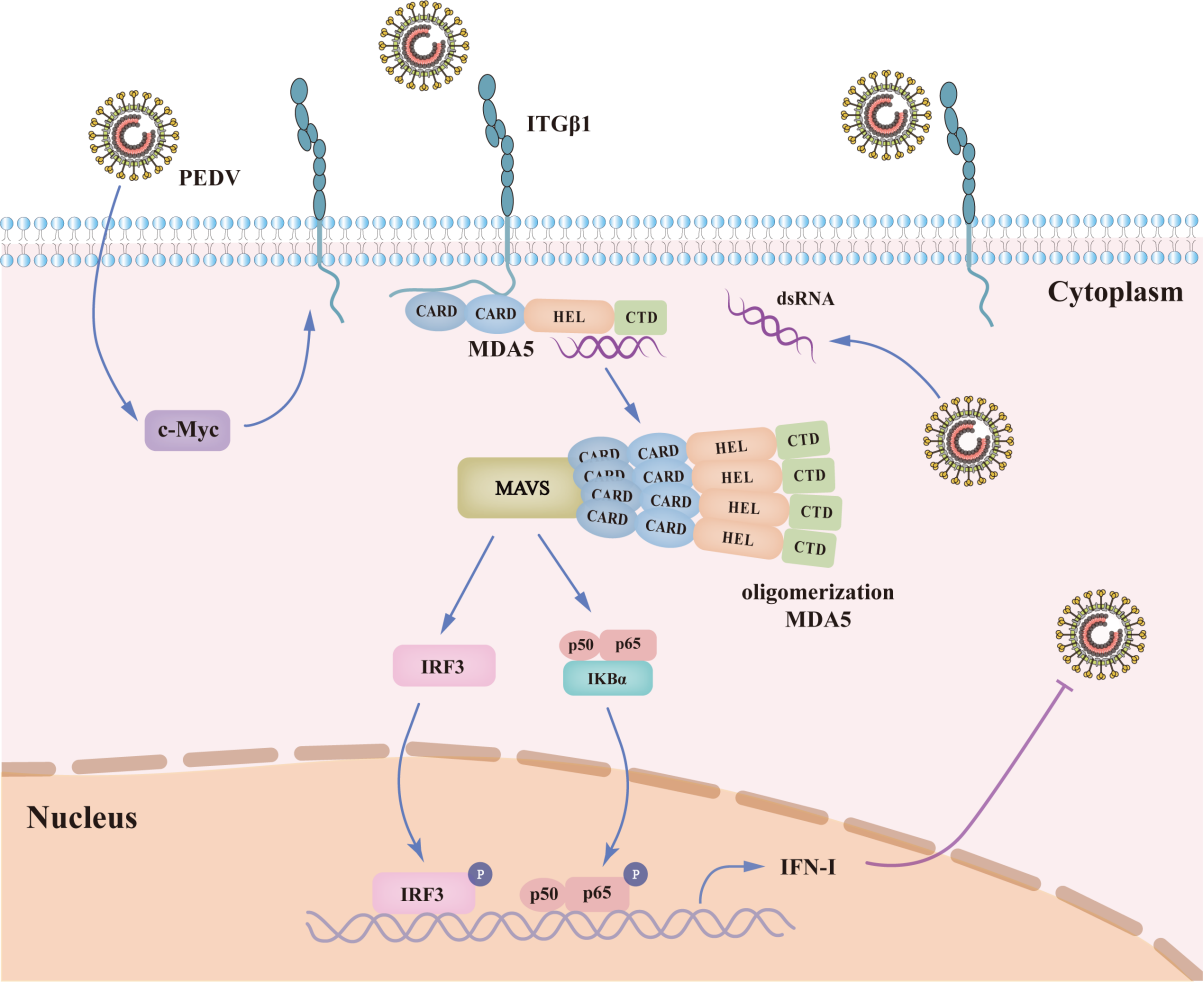
Mechanism of ITGβ1-mediated anti-PEDV activity. Following the infection of host cells by PEDV, the host cells activate the transcription factor c-Myc, which upregulates the expression of ITGβ1. The intracellular domain of ITGβ1 can interact with the 2CARD region of MDA5, promoting its oligomerization and subsequent activation. Activation of MDA5 initiates the phosphorylation and nuclear translocation of IRF3 and NF-κB, leading to the induction of IFN-β production.

Members of the RIG-I-like receptor (RLR) family, RIG-I and MDA5, serve as critical cytosolic sensors of viral RNA and play pivotal roles in initiating innate immune responses against RNA viruses (44). In the context of coronaviruses, MDA5 appears to dominate over RIG-I in recognizing viral dsRNA (45–47). Upon binding to viral RNA, MDA5 assembles along dsRNA via direct protein-protein interactions. This assembly facilitates oligomerization of the signaling domains (tandem CARDs) at the core of MDA5 filament, forming an elongated structure that activates MAVS (28, 29), thereby triggering downstream signaling cascades. In this study, we hypothesize that ITGβ1 may require MDA5’s dsRNA binding capacity to activate MDA5. To test this, we examined the effect of ITGβ1 ectopic expression on MDA5-mediated IFN-β production in the presence of poly(I:C). As anticipated, we observed a significant increase in IFN-β fluorescence. Conversely, dsRNA-induced IFN-β fluorescence intensity (luciferase reporter assay) increased markedly under ITGβ1 overexpression. Conversely, dsRNA-induced IFN-β transcriptional levels were significantly impaired in ITGβ1-knockout cell lines, and partial restoration of IFN-β transcription was observed upon ITGβ1 rescue. These findings indicated a functional interplay between ITGβ1 and MDA5 in the presence of dsRNA, with ITGβ1 acting as a positive regulator of MDA5-mediated antiviral responses by enhancing dsRNA-induced MDA5 activation. Given that MDA5 oligomerizes upon stimulation with dsRNA ligands, we further investigated the direct impact of ITGβ1 on MDA5 oligomerization. Strikingly, ectopic ITGβ1 expression robustly promoted MDA5 oligomerization and enhanced MDA5-driven IFN-β promoter activity. Notably, we identified the N-terminal 2CARD domains of MDA5 as essential for its interaction with ITGβ1. Mechanistically, ITGβ1 interacts with the 2CARD domains of MDA5 via its cytoplasmic tail region, suggesting that ITGβ1 may recruit MDA5 to increase its local concentration, thereby facilitating oligomerization (Fig. 10). Specifically, ITGβ1-mediated enhancement of MDA5 oligomerization occurs primarily through promoting 2CARD region oligomerization. Collectively, these results demonstrate that the host protein ITGβ1 potentiates MDA5 signaling activation, enabling host cells to mount rapid and robust antiviral responses against viral infection.

In summary, we report that the ITGβ1 functions as a novel host factor against PEDV by serving as a positive regulator of the host innate immune response. First, we demonstrated that ITGβ1 is essential for PEDV-induced antiviral responses, as its knockdown or knockout significantly attenuated IFN-β expression triggered by SeV or PEDV infection. Second, ITGβ1 enhanced IFN-β production in response to SeV or poly(I:C) stimulation. Mechanistically, ITGβ1 facilitates IFN-I production by promoting the phosphorylation and nuclear translocation of IRF3 and NF-κB, key transcription factors for interferon activation. Finally, ITGβ1 suppresses PEDV replication by inducing IFN-I expression through its ability to promote oligomerization of the MDA5 2CARD domains.

To our knowledge, this study provides the first evidence that an integrin protein, ITGβ1, acts as a critical activator of MDA5 in antiviral innate immunity. ITGβ1 orchestrates protective immune responses upon pathogen detection, highlighting its dual role in both PEDV infection and the regulation of MDA5-mediated antiviral signaling. These findings unveil a novel mechanism by which ITGβ1 targets MDA5 to modulate innate immunity, offering a promising therapeutic strategy for combating PEDV infection. Based on these findings, small-molecule agonists or antibodies targeting ITGβ1 could be developed to enhance the MDA5-mediated antiviral immune response in the early stage of infection, thereby inhibiting viral replication. Thus, as a key factor in MDA5 immune activation, ITGβ1 holds promise as a novel target for combating PEDV infection.

## MATERIALS AND METHODS

### Antibodies and reagents

The following antibodies and reagents were used in this study: monoclonal antibodies against p65 (8242T), phospho-p65 (3033S), IRF3 (11904S), phospho-IRF3 antibody (37,829T) and HA tag (3724S) were purchased from Cell Signaling Technology (CST). Anti-Glyceraldehyde-3-Phosphate Dehydrogenase (GAPDH) antibody (AC033) and anti-ITGβ1 antibody (A2217 and A21234) were obtained from Abclonal. Anti-β-actin antibody (60008-1), horseradish peroxidase (HRP)-conjugated anti-mouse IgG antibody (SA00001-1), and HRP-conjugated anti-rabbit IgG antibody (SA00001-2) were acquired from Proteintech Group. Anti-Flag tag antibody (M185-3L) was purchased from MBL. A monoclonal antibody against the PEDV N protein was generated in our laboratory (48). Furthermore, 4′6-diamidino-2-phenylindole (DAPI; C1002) was obtained from Beyotime, and the Dual-Glo Luciferase Assay System (DL101-01) was purchased from Vazyme Biotech.

### Cell culture

In this study, porcine kidney epithelial cells (LLC-PK1, obtained from the American Type Culture Collection), the ITGβ1-overexpressing LLC-PK1 cell line (LLC-PK1^ITGβ1^), and the ITGβ1-knockout LLC-PK1 cell line (LLC-PK1^ΔITGβ1^) were cultured in modified Eagle’s medium (MEM, catalog number 11095098, Life Technologies) supplemented with 10% fetal bovine serum (FBS, catalog number 10099141, Gibco). African green monkey kidney cells (Vero, also from the American Type Culture Collection), the ITGβ1-overexpressing Vero cell line (Vero^ITGβ1^) and HEK293T cells (American Type Culture Collection) were maintained in Dulbecco’s modified Eagle’s medium (DMEM, catalog number D6429, Sigma-Aldrich) supplemented with 10% FBS. All the aforementioned cell lines were incubated at 37°C in a humidified atmosphere containing 5% CO_2_.

### Virus infection

The PEDV strain FJzz1 (GenBank accession number MK288006) used in this study was isolated by our laboratory using Vero cells (20, 49). When Vero cells and LLC-PK1 cells were seeded in six-well plates reached a confluency of over 90%, they were thoroughly washed three times with phosphate-buffered saline (PBS). Subsequently, the cells were then infected with PEDV at an MOI of 0.01, 0.1, or 1. The infection medium consisted of DMEM or MEM without FBS but supplemented with 10 µg/mL trypsin. After incubation at 37°C for 1 h, the cells were washed three times with PBS to remove unbound viruses. Subsequently, the cells were cultured in FBS-free DMEM or MEM containing 10 µg/mL trypsin at 37°C. Virus titers were determined using the TCID_50_ method.

### Plasmid construction and RNA interference experiments

The porcine ITGβ1 gene was amplified using specific primers, and the resulting sequences were fused with HA or Flag tags at their C-termini before being cloned into the pcDNA3.1-CMV vector. The resulting constructs were designated as pcDNA3.1-ITGβ1-HA and pcDNA3.1-ITGβ1-Flag, respectively (Table 1). Similarly, the MDA5 gene was amplified and tagged with Flag or HA at its C-terminus. Followed by cloning into the pcDNA3.1-CMV vector. These constructs were named pcDNA3.1-MDA5-Flag and pcDNA3.1-MDA5-HA (Table 1). Additionally, the three functional domains of MDA5: 2CARD (amino acids 1–203), MDA5-ΔN domain (amino acids 204–1,025), and MDA5-ΔC domain (amino acids 1–575), were amplified and cloned into the pcDNA3.1-CMV vector with a C-terminal Flag tag using primers listed in Table 1. The resulting plasmids were termed pcDNA3.1-MDA5-2CARD-Flag, pcDNA3.1-MDA5-ΔN-Flag, and pcDNA3.1-MDA5-ΔC-Flag (Table 1). For RNA interference experiments, siRNA targeting the gene sequences of porcine ITGβ1 (GenBank accession number 397019) and monkey ITGβ1 (GenBank accession number 103238098) was designed and synthesized. The siRNA sequences are provided in Table 1. Vero cells and LLC-PK1 cells were seeded in 12-well plates and transiently transfected with the siRNA using Lipofectamine RNAi MAX transfection reagent (13778075; Thermo Fisher Scientific), following the manufacturer’s instructions. Thirty-six hours post-transfection, the cells were infected with PEDV, and qPCR analysis was performed using primers listed in Table 1.

**TABLE 1:** Primers and siRNA sequences used in the study

Primer name	Primer sequence (5′–3′)
ITGβ1-HA-F	ATGAATTTACAACTGATTTTCTGGATTG
ITGβ1-HA-R	TTAAGCGTAGTCTGGGACGTCGTATGGGTAGAGACCAGCTTTACGTCCATAG
ITGβ1-Flag-F	ATGAATTTACAACTGATTTTCTGGATTG
ITGβ1-Flag-R	TTACTTATCGTCGTCATCCTTGTAATCGAGACCAGCTTTACGTCCATAGTT
MDA5-Flag-F	ATGTCGAATGGGTATTCCACAGA
MDA5-Flag-R	CTACTTATCGTCGTCATCCTTGTAATCATCCTCATCACTAAATAAACAGCA
MDA5-HA -F	ATGTCGAATGGGTATTCCACAGA
MDA5-HA -R	CTAAGCGTAGTCTGGGACGTCGTATGGGTAATCCTCATCACTAAATAAACAGCA
MDA5-2CARD-Flag-F	ATGTCGAATGGGTATTCCACAGA
MDA5-2CARD-Flag-R	TCACTTATCGTCGTCATCCTTGTAATCTGAGCAATCAGAGCCTGTTAACTC
pcDNA3.1-MDA5-ΔN-Flag-F	ATGGAAAGCAATGCAGAGATTGA
pcDNA3.1-MDA5-ΔN-Flag-R	TCACTTATCGTCGTCATCCTTGTAATCATCCTCATCACTAAATAAACAGCA
pcDNA3.1-MDA5-ΔC-Flag-F	ATGTCGAATGGGTATTCCACAGA
pcDNA3.1-MDA5-ΔC-Flag-R	TCACTTATCGTCGTCATCCTTGTAATCAGTTCCAAAATCTGACATTGGACT
ITGβ1-1-558-3HA-F	CGTTTAAACTTAAGCTTGGTACCATGAATTTACAACTGATTTTCTGGATTGGACTG
ITGβ1-1-558-3HA-R	GCTGGATATCTGCAGAATTCGAAATTATCACACTCGCAGAATTTGCC
ITGβ1-465-751-3HA-F	CGTTTAAACTTAAGCTTGGTACCATGGAATGCCAGAGCGAGGGCATC
ITGβ1-465-751-3HA-R	GCTGGATATCTGCAGAATTCCCAAATGAGCAGCAGTGC
ITGβ1-729-798-3HA-F	CGTTTAAACTTAAGCTTGGTACCATGATTATTCCAATTGTAGCTGGTG
ITGβ1-729-798-3HA-R	GCTGGATATCTGCAGAATTCTTTTCCCTCATACTTCGGATTG
Pig-si-ITGβ1-F	GGUCCAGACAUUAUUCCAATT
Pig-si-ITGβ1-R	UUGGAAUAAUGUCUGGACCTT
Monkey-si-ITGβ1-F	GCAGCACAGA UGAAGUUAATT
Monkey-si-ITGβ1-R	UUAACUUCAUCUGUGCUGCTT
Pig-si-TRM1-F	GGACAAAGCUGAAGUGAAATT
Pig-si-TRM1-R	UUUCACUUCAGCUUUGUCCTT
Pig-si-TFEB-F	GGAGGUUCAACAUCAAUGATT
Pig-si-TFEB-R	UCAUUGAUGUUGAACCUCCTT
Pig-si-TFE3-F	GUGGAUUAUAUCCGCAAAUTT
Pig-si-TFE3-R	AUUUGCGGAUAUAAUCCACTT
Pig-si-c-Myc-F	CAAGGUAGUUAUCCUUAAATT
Pig-si-c-Myc-R	UUUAAGGAUAACUACCUUGTT
NC-F	UUCUCCGAACGUGUCACGUTT
NC-R	ACGUGACACGUUCGGAGAATT
qPCR-PEDV-N-F	CGCAAAGACTGAACCCACTAAC
qPCR-PEDV-N-R	TTGCCTCTGTTGTTACTTGGAGAT
qPCR-pig-GAPDH-F	GATTCCACCCACGGCAAGTTCC
qPCR-pig-GAPDH-R	AGCACCAGCATCACCCCATTTG
qPCR-monkey-GAPDH-F	TGACATCAAGAAGGTGGTGAAGCAG
qPCR-monkey-GAPDH-R	GTGTCGCTGTTGAAGTCAGAGGAG

### RNA extraction and qRT-PCR

Total RNA was extracted from PEDV-infected Vero or LLC-PK1 cells using the RNeasy Mini Kit (QIAGEN, 74104) according to the manufacturer’s instructions. The extracted RNA was then reverse transcribed into cDNA utilizing the Revert Aid First Strand cDNA Synthesis Kit (Thermo Fisher Scientific, K1622). The resulting cDNA was used as a template for qPCR analysis, which was performed using SYBR Green PCR Mix (Vazyme Biotech, q711-03) on the Light Cycler system. Specific primers used for all qPCR reactions in this study are listed in Table 1. The GAPDH gene was used as an internal reference gene for normalization of qPCR results.

### Western blotting

Vero cells and LLC-PK1 cells, either infected or mock-infected with PEDV, were harvested at the indicated time points and washed with ice-cold PBS. The cells were then lysed on ice for 10 min using RIPA lysis buffer (Thermo Fisher Scientific, 89901) supplemented with protease inhibitors (Bimake, B14001) and phosphatase inhibitors (Bimake, B15001). After lysis, the samples were centrifuged at 12,000 rpm for 10 min at 4°C. The supernatant was collected and mixed with 5× SDS PAGE loading buffer, followed by heating at 95°C for 10 min to denature proteins. The denatured proteins were separated by SDS-PAGE electrophoresis and subsequently transferred onto a 0.2 µm nitrocellulose membrane (GE Healthcare, 10600001) for Western blotting. The transferred membrane was blocked with 5% skim milk in TBST for 2 h at room temperature and washed three times with TBST. It was then incubated with the appropriate mouse or rabbit primary antibody for 1 h at room temperature, followed by three washes with TBST. Next, the membrane was incubated with an HRP-conjugated anti-rabbit IgG or anti-mouse IgG secondary antibody for 1 h. After three additional washes with TBST, protein signals were detected using enhanced chemiluminescence (Share-bio, SB-WB012).

### Dual-luciferase reporter assay

HEK293T cells were seeded in 24-well plates and grown to 80% confluence. The cells were then transfected with luciferase reporter plasmids (IFN-β-Luc, NF-κB-Luc, IRF3-Luc, pRL-TK, and ITGβ1-Luc) along with the specified expression plasmids or empty vector controls using Lipofectamine 2000 (Invitrogen, 11668019). Twenty-four hours post-transfection, the cells were either stimulated with SeV for 12 h or harvested directly, depending on the experimental design. Luciferase activity was measured using the Dual-Glo Luciferase Assay System (Vazyme, DL101-01) according to the manufacturer’s instructions, with Renilla luciferase activity value serving as an internal control for normalization.

### Immunofluorescence assay

HEK293T cells were seeded onto coverslips in six-well plates and transfected with pcDNA3.1-ITGβ1-HA or an empty vector for 24 h. The cells were then either mock-infected or infected with SeV for 12 h. After infection, the cells were fixed with 4% paraformaldehyde for 20 min and permeabilized using 0.5% Triton X-100 for 10 min at room temperature. Following three washes with PBS, the cells were blocked with PBS containing 5% bovine serum albumin for 1 h at 37°C. The cells were then incubated with rabbit anti-IRF3 antibody (1:500), rabbit anti-p65 antibody (1:500), and mouse anti-HA antibody (1:1,000) at 37°C for 1 h. After an additional three washes with PBS, the cells were incubated in the dark with Alexa Fluor 594-conjugated goat anti-mouse IgG and Alexa Fluor 488-conjugated goat anti-rabbit IgG for 1 h. The nuclei were then stained with DAPI for 15 min, and images were captured using a Zeiss laser confocal microscope.

### Co-IP assay

HEK293T cells were transfected with the specified plasmids in six-well plates. Twenty-four hours post-transfection, the cells were lysed on ice for 30 min using NP-40 cell lysis buffer (Life Technologies, FNN0021) supplemented with protease and phosphatase inhibitors. After centrifugation at 12,000 rpm for 10 min at 4°C, a portion of the supernatant from the cell lysate was collected for whole-cell extract analysis. The remaining supernatant was incubated with antibody-coated magnetic beads at room temperature for 1 h. The beads were then washed three times with 0.02% PBST. Following washing, the magnetic beads containing the immunoprecipitation complex were resuspended in SDS-PAGE loading buffer, heated for 10 min, and subjected to SDS-PAGE. The separated proteins were transferred onto 0.2 µm nitrocellulose membranes for Western blotting. The membranes were incubated with the appropriate antibodies and the signals were detected using enhanced chemiluminescence.

### Detection of protein oligomerization

HEK293T cells were harvested at 24 h post-transfection and washed three times with PBS. Protein oligomerization was analyzed using two approaches: (i) native polyacrylamide gel electrophoresis (Native-PAGE): cells were lysed in native lysis buffer (Proteintech, PR20001) supplemented with protease inhibitors. Cell lysates were centrifuged at 12,000 rpm for 10 min at 4°C to collect proteins, which were then mixed with 4× native buffer (0.25 M Tris-HCl, pH 6.8, 40% glycerol, 4% sodium deoxycholate, and 0.005% bromophenol blue) for native PAGE. Following electrophoresis, immunoblotting was performed. (ii) DSS cross-linking: cells were resuspended in PBS, and DSS was added to a final concentration of 5 mM. After incubation at room temperature for 30 min to cross-link oligomers, 50 mM Tris-HCl (final concentration) was added to terminate the reaction for 10 min. Cells were centrifuged at 1,000 rpm for 5 min at 4°C, then lysed in RIPA lysis buffer containing protease inhibitors. Proteins were collected by centrifugation, subjected to SDS-PAGE, and analyzed by immunoblotting after electrophoresis (50).

### Statistical analysis

Statistical analysis was performed using GraphPad Prism 6 (GraphPad Software, USA). Comparisons were made using Student’s *t*-test. *P* < 0.05 (*), *P* < 0.01 (**), *P* < 0.001 (***), *P* < 0.0001 (****), and ns indicates not significantly different. Data are the means of at least three independent experiments.

## Data Availability

All relevant data are within the article.

## SUPPLEMENTAL MATERIAL

Figure S1 jvi.01553-25-s0001.tif Localization of ITGβ1 in HEK293T cells.

Supplemental legend jvi.01553-25-s0002.docx Legend for Fig. S1.

ASM does not own the copyrights to Supplemental Material that may be linked to, or accessed through, an article. The authors have granted ASM a non-exclusive, world-wide license to publish the Supplemental Material files. Please contact the corresponding author directly for reuse.
