# Supplementary material for: The integrin protein ITGβ1 effectively suppresses porcine epidemic diarrhea virus replication through facilitating MDA5 oligomerization and subsequent activation of the type I interferon signaling pathway
Source: J Virol. 2025 Oct 17;99(11):e01553-25. doi: 10.1128/jvi.01553-25 (PMC12645938; doi:10.1128/jvi.01553-25)
Supplement: Supplemental legend — Legend for Fig. S1. [file jvi.01553-25-s0002.docx]

Figure S1. Localization of ITGβ1 in HEK293T Cells. Subsequent to the overexpression of ITGβ1 in HEK293T cells, the fixed cells were either permeabilized with Triton X-100 or remained non-permeabilized. ITGβ1 was labeled via indirect immunofluorescence, and its subcellular localization was visualized under a Zeiss laser confocal microscope.
